# A Thermosensitive Bi‐Adjuvant Hydrogel Triggers Epitope Spreading to Promote the Anti‐Tumor Efficacy of Frameshift Neoantigens

**DOI:** 10.1002/advs.202306889

**Published:** 2024-02-02

**Authors:** Yaohua Ke, Kai Xin, Yaping Tao, Lin Li, Aoxing Chen, Jingyi Shao, Junmeng Zhu, Dinghu Zhang, Lanqi Cen, Yanhong Chu, Lixia Yu, Baorui Liu, Qin Liu

**Affiliations:** ^1^ The Comprehensive Cancer Centre Nanjing Drum Tower Hospital Affiliated Hospital of Medical School Nanjing University 321 Zhongshan Road Nanjing 210008 China; ^2^ Department of Oncology Nanjing Drum Tower Hospital Clinical College of Nanjing University of Chinese Medicine 321 Zhongshan Road Nanjing 210008 China; ^3^ Zhejiang Cancer Hospital Hangzhou Institute of Medicine (HIM) Chinese Academy of Sciences Hangzhou 310022 China

**Keywords:** cGAS‐STING agonist, frameshift neoantigens, thermosensitive hydrogel, TLR7/8 agonist, tumor immunotherapy

## Abstract

Tumor‐specific frameshift mutations encoding peptides (FSPs) are highly immunogenic neoantigens for personalized cancer immunotherapy, while their clinical efficacy is limited by immunosuppressive tumor microenvironment (TME) and self‐tolerance. Here, a thermosensitive hydrogel (FSP‐RZ‐BPH) delivering dual adjuvants R848 (TLR7/8 agonist) + Zn^2+^ (cGAS‐STING agonist) is designed to promote the efficacy of FSPs on murine forestomach cancer (MFC). After peritumoral injection, FSP‐RZ‐BPH behaves as pH‐responsive sustained drug release at sites near the tumor to effectively transform the immunosuppressive TME into an inflammatory type. FSP‐RZ‐BPH orchestrates innate and adaptive immunity to activate dendritic cells in tumor‐draining lymph nodes and increase the number of FSPs‐reactive effector memory T cells (*T*_EM_) in tumor by 2.9 folds. More importantly, these *T*_EM_ also exhibit memory responses to nonvaccinated neoantigens on MFC. This epitope spreading effect contributes to reduce self‐tolerance to maintain long‐lasting anti‐tumor immunity. In MFC suppressive model, FSP‐RZ‐BPH achieves 84.8% tumor inhibition rate and prolongs the survival of tumor‐bearing mice with 57.1% complete response rate. As a preventive tumor vaccine, FSP‐RZ‐BPH can also significantly delay tumor growth. Overall, the work identifies frameshift MFC neoantigens for the first time and demonstrates the thermosensitive bi‐adjuvant hydrogel as an effective strategy to boost bystander anti‐tumor responses of frameshift neoantigens.

## Graphical abstract

This study presents the development of a novel thermosensitive hydrogel featuring the integration of neoantigens and dual adjuvants. The intricate design of this hydrogel, facilitated by Zn2+, activates both the TLRs and cGAS‐STING signaling pathway. The resulting synergistic effect successfully amplifies the specificity of the anti‐tumor immune response to neoantigens and elicits a potent bystander effect.

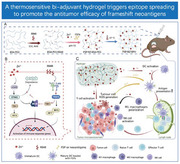

## 1 Introduction

Immunogenic tumor‐specific antigens are initiation of anti‐tumor immunity and the key to T cell‐mediated immune response.^[^ ^1^ ^]^ Neoantigens are highly immunogenic and regarded as the most promising tumor antigens, which are derived from non‐synonymous mutations in the genome, only expressed in tumor cells and able to escape the negative selection of thymus.^[^ ^2^ ^]^ Non‐synonymous mutations mainly include point mutations, frameshift mutations, splice‐site mutations and fusion mutations, and a higher burden of non‐synonymous mutations can enhance the immunogenicity of neoantigens.^[^ ^3^ ^]^ Point mutations neoantigens are derived by single‐nucleotide variants (SNVs), including KRAS G12V/D, p53 R175H, etc.^[^ ^4^ ^]^ Differently, frameshift peptides (FSPs) come from insertion or deletion (INDEL) of base pair in the genome. Since all downstream amino acid sequences after “INDEL” have been altered, FSPs contain a higher burden of nonsynonymous mutations than SNV‐derived neoantigens.^[^ ^5^ ^]^ At present, SNV‐derived neoantigens have achieved good clinical effects in melanoma, which contains the largest number of SNVs^[^ ^2^, ^6^ ^]^ In lung cancer and bladder cancer, immune checkpoint inhibitors (ICIs) have been proved to enhance the efficacy of SNV‐derived neoantigen vaccines.^[^ ^6^, ^7^ ^]^ Unfortunately, for tumors with low mutation burden such as clear‐cell renal‐cell carcinoma, sarcoma and gastric cancer, SNVs‐derived neoantigens are difficult to achieve clinical efficacy.^[^ ^8^ ^]^ For these tumors, targeting FSPs may be a better option to develop neoantigen vaccines. In a recent clinical study, involving a cohort of six individuals diagnosed with gastric cancer and exhibiting ASTE1 mutations, a notable finding emerged: the identification of the R632Gfs^*^33 frameshift mutation in a substantial majority, specifically detected in five out of the six patients, constituting an impressive proportion of 83.3%. However, in another comprehensive study involving 727 patients diagnosed with gastric cancer and exhibiting TP53 mutations, the findings revealed that 80 individuals (11.0%) presented frameshift mutations, while 312 patients (42.9%) displayed conversion mutations, and 335 cases (46.1%) showcased transversion mutations.^[^ ^9^ ^]^ While the occurrence rates of frameshift mutations in genes or genetic pathways linked to gastric cancer can vary significantly, existing research has consistently indicated that the targeting frameshift mutations holds greater promise for yielding clinical benefits compared to SNVs mutations in several tumors. This assertion is particularly evident when considering their combination with ICIs, a synergy that has been validated through numerous studies in the field.^[^ ^5^, ^10^ ^]^

The process of neoantigen‐specific anti‐tumor immune response mainly involves the antigen‐presenting cells (APCs) represented by dendritic cells (DCs) phagocytosing neoantigens and presenting them to T cells for recognition, thereby generating neoantigen‐reactive T cells (NRTs).^[^ ^11^ ^]^ However, it is difficult for NRTs to fully function in the immunosuppressive tumor microenvironment (TME), and under the selection pressure from the immune system, tumor cells are prone to novel mutations and neoantigens, resulting in self‐tolerance to further limit the tumor killing effect of NRTs.^[^ ^12^ ^]^ The strategy of simultaneously activating adaptive and innate immunity has been shown to reserve the immunosuppressive TME and maximize the function of NRTs.^[^ ^13^ ^]^ As exogenous substances, adjuvants targeting innate immunity can recognize pattern recognition receptors (PRRs) on recipient cells, activate downstream toll‐like receptors (TLRs), nod‐like receptors (NLRs), cGAS‐STING and other signaling pathways to transform recipient cells (DCs, natural killer cells, macrophages and so on) from a calm state to be pro‐inflammatory, thus generating a robust anti‐tumor immune response.^[^ ^14^ ^]^ Currently, there are a number of clinical trials based on innate immune adjuvants, in which TLRs agonists (including CpG, R848 and poly‐ICLC, etc.) and STING agonists (cyclic dinucleotides, metal ions, etc.) are most notable.^[^ ^15^ ^]^ Due to the convenience and powerful effect, the immunotherapy using Mn^2+^, Zn^2+^, and other metal ions as cGAS‐STING agonists has recently attracted much attention.^[^ ^16^ ^]^ Besides, Zn^2+^ can also induce the generation of reactive oxygen species (ROS) to directly kill tumor cells.^[^ ^17^ ^]^ Interestingly, TLRs agonists can exert an inhibitory influence on the degradation process of STING molecules by facilitating the upregulation of NF‐κB expression. This process ultimately enhances the effectiveness of STING agonists,^[^ ^18^ ^]^ which suggests TLRs agonists can synergize with cGAS‐STING agonists to promote anti‐tumor immunity.

Activation of innate immunity can enhance the response of adaptive immunity, but large doses of TLRs or cGAS‐STING agonists are prone to severe systemic inflammatory side effects due to their non‐specificity. Similarly, ROS also shows duality in tumor progression. In TME, ROS exhibits anti‐tumor effects, but in normal tissue, it can cause cancerogenic mutations and death.^[^ ^19^ ^]^ In the pursuit of harnessing the anti‐tumor effects of adjuvants while mitigating potential toxicity, meticulous consideration must be given to dose selection and target accuracy. Conventional safety administration practices involve fractionating a single dose into multiple smaller doses, with sustained‐release carriers emerging as an advantageous approach to simultaneously reduce both the individual dose and the frequency of administrations. Hydrogel, owing to its commendable in vivo biocompatibility, stands out as an exemplary sustained‐release drug carrier. Furthermore, a hydrogel can be tailored as a TME‐responsive drug carrier, thereby ensuring the safe and effective activation of anti‐tumor immunity.^[^ ^20^ ^]^

**Scheme 1:**
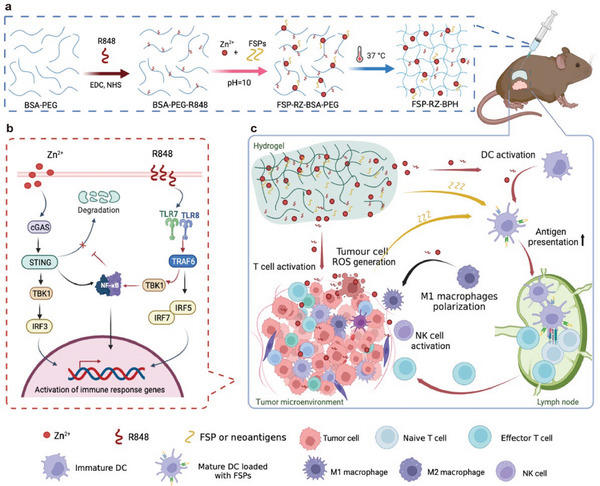
Schematic illustration of the thermosensitive bi‐adjuvant hydrogel (FSP‐RZ‐BPH). a) Formation of FSP‐RZ‐BPH. The schematic diagram shows that R848, Zn^2+^ and FSPs are separately loaded on the BSA‐PEG to form a hydrogel skeleton, and its aqueous solution can form a hydrogel at 37 °C. b) Mechanism of synergistic proinflammatory effect of R848 + Zn^2+^ (RZ). R848 is a TLR7/8 agonist, which inhibits the degradation of STING by activating NF‐κB molecules, thereby promoting the Zn^2+^‐activated cGAS‐STING pathway. c) Schematic diagram to illustrate the immune response induced by FSP‐RZ‐BPH in vivo. Combined application of RZ can not only directly activate intratumoral reactive oxygen species (ROS), but also have pro‐inflammatory effects on DCs, T cells, NK cells and macrophages. Activation of DCs promotes the presentation of FSPs, generating a large number of effector T cells in the lymph nodes, which eventually leads to the increase of tumor‐infiltrating NRTs to kill tumor cells. In particular, sufficient tumor neoantigens released by ROS‐induced tumor cell death and the sustained immune activation of FSP‐RZ‐BPH endows T cells epitope spreading to maintain long‐term anti‐tumor immune responses.

In this work, we designed an intelligent thermosensitive hydrogel FSP‐RZ‐BPH to deliver dual adjuvants (R848 + Zn^2+^, RZ) and FSPs for the treatment of MFC (**Scheme** **1**). We first screened the FSPs on MFC and verified their immunogenicity. Afterward, we systematically test the synergetic pro‐inflammatory effects of R848 (TLR7/8 agonist) combined with Zn^2+^ (cGAS‐STING agonist) on variant immune cells and the ROS on MFC cells. FSP‐RZ‐BPH is an injectable thermosensitive hydrogel engineered by Zn^2+^, which greatly improves the performance of the hydrogel, making it gelatinize and degrade faster, and exhibit a pH‐responsive drug sustained‐release effect. FSP‐RZ‐BPH can fully orchestrate innate and adaptive immunity to activate local anti‐tumor immune response, and can trigger epitope spreading effect to reduce self‐tolerance. In the MFC suppressive and preventive model, FSP‐RZ‐BPH can both significantly inhibit tumor growth and prolong the survival of tumor‐bearing mice. To the best of our knowledge, this is the first exploration of delivering FSPs by thermosensitive bi‐adjuvant hydrogel to achieve long‐lasting anti‐tumor immunity.

## 2 Results

### 2.1 Screening and Validation of FSPs

**Figure 1:**
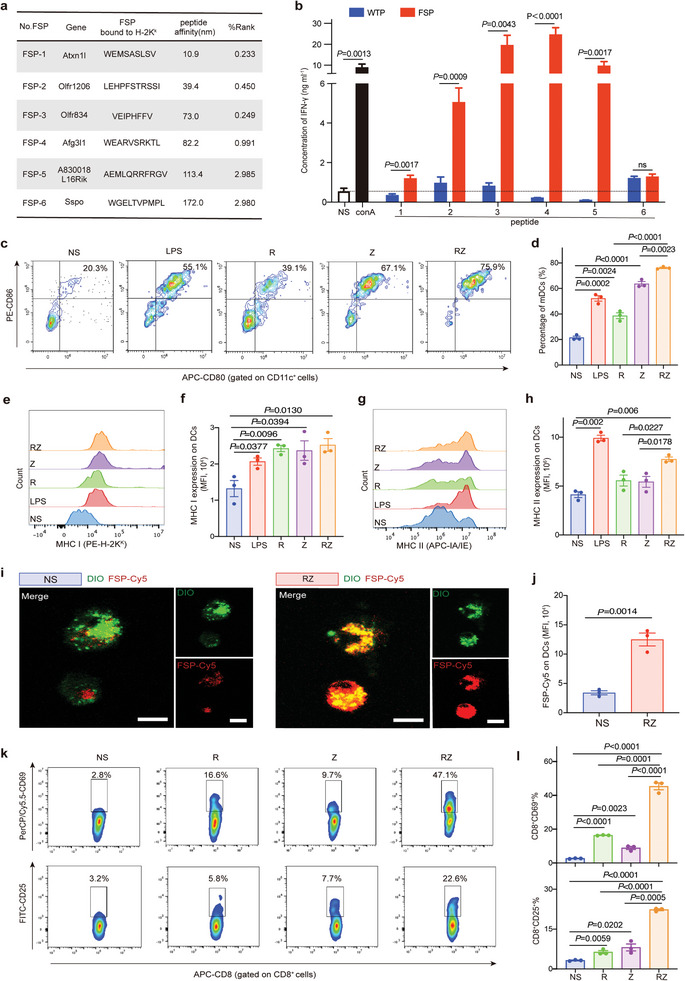
Identification of FSPs and the pro‐inflammatory effect of R848 + Zn^2+^ (RZ). a) Amino acid sequence of FSPs and their affinity to H‐2K^K^. b) Concentration of IFN‐γ secreted by splenocytes of 615 mice stimulated by FSPs or wild‐type peptides (WTPs). NS represented normal saline, and conA represented concanavalin A. c) Representative flow cytometry images of mature DCs (mDCs, CD11c^+^CD80^+^CD86^+^) after co‐incubation with RZ in vitro for 48 h. d) The percentage of mDCs (n = 3). e) Representative flow cytometry images of MHC I expression on DCs after co‐incubation with RZ in vitro for 48 h. f) The levels of MHC I expression on DCs (n = 3). g, Representative flow cytometry images of MHC II expression on DCs after co‐incubation with RZ in vitro for 48 h. h) The levels of MHC II expression on DCs (n = 3). i) Confocal images of DCs after co‐incubation with FSP‐Cy5 and RZ in vitro for 48 h (The scale bar is 10 µm). j) The levels of FSP‐Cy5 expression on DCs after co‐incubation with FSP‐Cy5 and RZ in vitro for 48 h (n = 3). k) Representative flow cytometry images of CD8^+^CD25^+^ T cells and CD8^+^CD69^+^ T cells after co‐incubation with RZ in vitro for 48 h. l, The percentage CD8^+^CD25^+^ T cells and CD8^+^CD69^+^ T cells (n = 3). The error bars represented mean ± SEM. *P*‐values were calculated by two‐tailed unpaired Student's t‐tests. ns represented p > 0.05.

MFC‐targeting FSPs were identified with whole‐exome sequencing (WES) and neoantigen prediction tools, and 9–11 mer mutant peptides with strong binding affinity to H‐2K^k^(615 mouse HLA type)were considered potentially effective neoantigens. We selected the top six peptides as MFC‐targeting FSPs according to their affinity (**Figure** **1**a). We investigated the immunogenicity of FSPs in vitro using splenocytes from 615 mice. Over seven days, splenocytes were tested for IFN‐γ levels after peptide pulses for three times. Compared with the wild‐type peptides (WTPs), FSP 1–5 significantly stimulated higher levels of IFN‐γ. The concentration of IFN‐γ induced by FSP‐3,4,5 increased more than tenfold, and FSP‐4 got the best stimulated effect, whose concentration of IFN‐γ reaching 2461.7 pg ml^−1^, 114‐fold that of WTP‐4 (21.6 pg ml^−1^) (Figure 1b). As mentioned above, frameshift mutations have a higher burden of non‐synonymous mutations, and FSPs are more immunogenic than SNVs‐induced neoantigens in the same number of peptides. We used the same method to test the levels of IFN‐γ stimulated by SNVs‐induced MFC neoantigens (MFCs),^[^ ^21^ ^]^ and it could be seen that the concentration of IFN‐γ increased no more than fivefold compared with NS (Figure S1, Supporting Information), which indicated that FSPs were better targets for personalized neoantigen vaccines on gastric cancer.

### 2.2 Zn^2+^ Synergizes with R848 to Turn TME Toward Pro‐Inflammatory

Co‐delivery with immune adjuvants to enhance the effect of neoantigen vaccines has gradually become a consensus.^[^ ^22^ ^]^ However, a single adjuvant often lacks sufficient strength and can't produce broad effects on multiple immune cells or tumor cells in TME. Here, we demonstrated that RZ exerted a powerful synergistic effect in the pro‐inflammatory transformation of TME containing DCs, macrophages, T cells, and MFC cells. Safe concentrations of Zn^2+^ and R848 were tested using human umbilical vein endothelial cells (HUVECs) to ensure that the doses of all subsequent experiments were within the safe range. The concentration of R848 used in all cell experiments was 3 µg mL^−1^, and the concentration of Zn^2+^ was 50 µm (Figure S2, Supporting Information). Using LPS as a positive control, we tested the activation effect of RZ on bone marrow‐derived DCs (BMDCs). The results confirmed that both Zn^2+^ and R848 could promote the activation of DCs. The best activation effect was obtained when they were used in combination (RZ), which was manifested by an increase in the ratio of CD80 and CD86 expression on the DCs from 21.5% in NS to 76.1% (Figure 1c,d). RZ also significantly augmented the concentrations of TNF‐α, IL‐12(p70), IL‐6, and IL‐10 in the supernatant of BMDCs subsequent to stimulation with RZ, providing additional evidentiary support for this conclusion (Figure S3, Supporting Information). DCs phagocytose foreign antigens and present the endocytosed antigens to the surface, where they are recognized by T cells in the form of peptide‐MHC, and the expression of MHC on DCs is an indicator of their antigen presenting capacity.^[^ ^23^ ^]^ As shown in Figure 1e–h, both Zn^2+^ and R848 could increase the levels of MHC I and MHC II, and showed a significant synergistic effect on expression of MHC II, increasing the number of FSPs presented on the surface of DCs by 3.7 fold (Figure 1i,j).

We then verified the effect of RZ on T cells by testing CD69 and CD25, which are markers of early and late T cells activation, respectively. For CD8^+^ T cells, Zn^2+^ and R848 stimulation increased the proportion of CD69 expression by 6.1 fold (16.4%) and 3.3 fold (8.9%) compared to NS (2.7%). Surprisingly, the stimulatory effect of RZ was greatly enhanced to 17.0 fold (45.3%) (Figure 1k,l). Besides, the expression of CD25^+^ on CD8^+^ T cells, CD69^+^ and CD25^+^ on CD4^+^ T cells also showed similar trends, reflecting the broad and powerful co‐stimulatory effects of Zn^2+^ combined with R848 on T cells (Figure S4a,b, Supporting Information).

In addition to DCs and T cells, other components of the TME can also influence the immune response. Tumor‐associated macrophages (TAM) are dominated by immunosuppressive M2 macrophages. Reducing M2‐TAM and increasing immune‐promoting M1‐TAM can improve the effect of immunotherapy. Using RAW264.7 cells, we tested the effect of R848 and Zn^2+^ on the immune status of macrophages. It can be found that both R848 and Zn^2+^ could increase the proportion of M1 macrophages, in which R848 was better than Zn^2+^. R848 could also reduce the proportion of M2 macrophages (Figure S4c–f, Supporting Information). RZ could reverse the suppressive state of macrophages to the greatest extent, increasing the ratio of M1/M2 from 15.4% to 91.7% (Figure S4g, Supporting Information). In addition to the effect on immune cells, Zn^2+^ has been proved to induce ROS production in tumor cells to directly kill tumor cells, and we wondered whether R848 could promote this effect. As shown in Figure S4h,i, Supporting Information, although R848 hardly increased ROS, the ROS produced by R848 + Zn^2+^ was greatly increased than that of Zn^2+^, indicating that R848 could enhance the ROS induction of Zn^2+^.

### 2.3 Construction and Characterization of FSP‐RZ‐BPH

**Figure 2:**
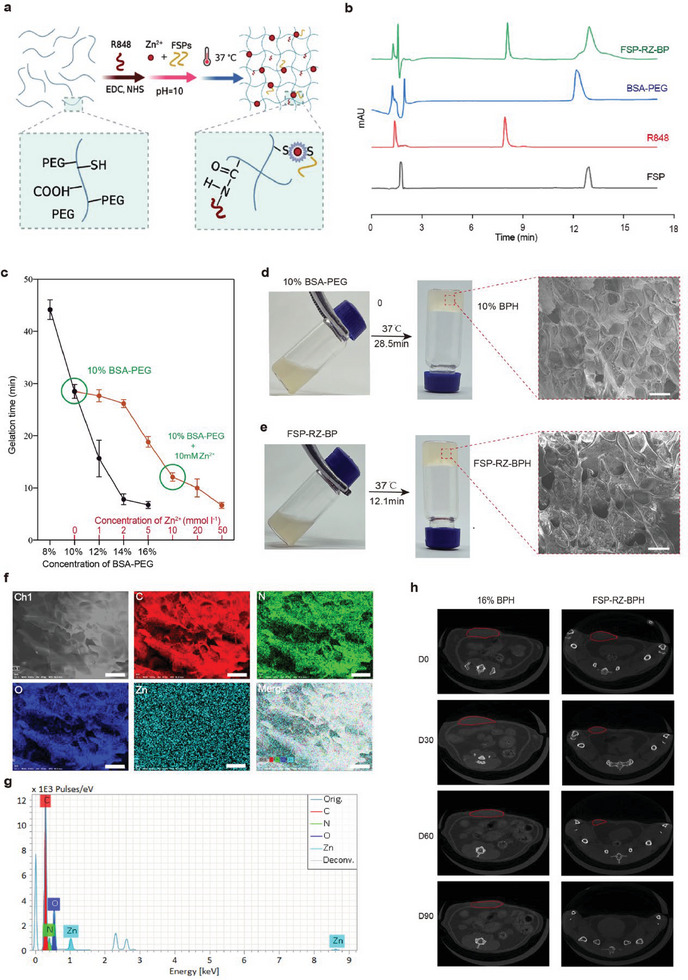
Construction and characterization of FSP‐RZ‐BPH. a) Schematic diagram to show the construction of FSP‐RZ‐BPH. b) HPLC spectra of FSP, R848, BSA‐PEG and FSP‐RZ‐BPH. c) Gelation time of BSA‐PEG hydrogel in different concentration of BSA‐PEG and Zn^2+^ (n = 3). d) Formation of 10% BSA‐PEG hydrogel (BPH) and its scanning electron microscope (SEM) images. e) Formation of FSP‐RZ‐BPH and its scanning SEM images. f) The C, N, O and Zn element mapping in FSP‐RZ‐BPH by SEM with energy‐dispersive X‐ray spectroscopy (SEM‐EDS). g) EDS spectrum of SEM‐EDS mapping for FSP‐RZ‐BPH. h) Computed tomography (CT) images of 615 mice on D0, D30, D60 and D90 after injecting 16% BSA‐PEG hydrogel (16% BPH) or FSP‐RZP‐BPH into the lower right abdomen. The hydrogel was circled in red.

Bovine serum albumin (BSA) and poly(ethylene glycol) (PEG) are able to form a thermosensitive BSA‐PEG hydrogel (BPH) that is mild to the human body and has been used as a carrier of chemotherapy drugs.^[^ ^24^ ^]^ PEG holds FDA approval for clinical use, while BSA serves as a model protein for pharmacokinetic testing and is widely employed as a drug carrier in animal experiments, known for its minimal propensity to induce foreign body reactions. Additionally, as a congener with a similar composition, BSA can readily be substituted by FDA‐approved human serum albumin (HSA) in subsequent clinical trials. Moreover, the abundance of active chemical groups in the side chains of BSA, such as ─COOH, ─NH_2_, and ─SH, provides convenient sites for further drug coupling without compromising its gel‐forming ability. The disadvantage of BPH is that the gelation time is too long at 37 °C that most of the drugs will release before the hydrogel forms. Increasing the concentration of BSA‐PEG can speed up the gelation time, but the resulting hydrogel is difficult to degrade in vivo. The BPH formed by subcutaneous injection of 16% BSA‐PEG has been shown not to degrade after 200 days, which hindered its clinical application.^[^ ^24b^ ^]^ We constructed a thermosensitive hydrogel named FSP‐RZ‐BPH to shorten the gelation time, which could also exhibit pH‐responsive drug release. Through amino‐carboxyl dehydration condensation, R848 could be linked to the side chain of BSA, and the encapsulation efficiency reached 17.2%. The formation of the ZN‐S coordination bond could connect FSPs to ‐SH of BSA, and the encapsulation efficiency reached 26.5% (**Figure** **2**a,b). Prior to this, all FSPs amino acid sequences were modified with cysteine (containing ‐SH) at the C‐terminus. The addition of Zn^2+^ greatly changed the gelation process of BPH. Under 10% BSA‐PEG, increasing the concentration of Zn^2+^ can effectively shorten the gelation time (Figure 2c). When the Zn^2+^ reached 10 mm, the gelation time can be shortened from 28.5 to 12.1 min, and we chose not to continually increase the concentration of Zn^2+^ in consideration of safety. Due to the reduced concentration of BSA‐PEG, the time for the hydrogel to completely degrade in vivo was greatly shortened to ≈90 days (Figure 2h). Finally, 10% BSA‐PEG was successfully engineered to be FSP‐RZ‐BPH with FSPs and the dual adjuvants. There were 1 mg FSPs for each peptide, 1.32 mg Zn^2+^ and 500 µg R848 in FSP‐RZ‐BPH, and some of these drugs were chemically linked to BSA as described above, while others existed in the hydrogel in a free form. We also conducted rheological assessments of BPH and FSP‐RZ‐BPH, as illustrated in Figure S5, Supporting Information. The findings indicate that the solution‐gelation transition for BPH and FSP‐RZ‐BPH precursor solutions occurs at ≈33 and 35 °C, respectively. This property enables the maintenance of precursor solutions in a liquid state suitable for injection at room temperature, followed by subsequent hydrogel formation at the physiological temperature of 37 °C. The observed increase in the storage modulus of FSP‐RZ‐BPH corresponds to a 2 °C reduction in the gelation temperature, aligning with the shortened gelation time of BPH in the presence of Zn^2+^, as depicted in Figure 2c. Dynamic oscillation strain scanning reveals a broad linear viscoelastic region for the hydrogel in the low‐strain domain. At 37 °C, both the storage modulus and loss modulus of the two hydrogels exhibit relative stability with an increase in angular frequency. Furthermore, the viscosity of the hydrogels markedly decreases with rising shear rates, indicating a beneficial shear‐thinning property for injectable drug delivery systems. On the SEM image, BPH and FSP‐RZ‐BPH both showed pore sizes ranging from 20 to 50 µm (Figure 2d,e). We further confirmed the existence of Zn element in FSP‐RZ‐BPH using EDS‐mapping, and Zn element was uniformly distributed throughout the hydrogel (Figure 2f,g).

### 2.4 pH‐Responsive Sustained Drug Release of FSP‐RZ‐BPH

**Figure 3:**
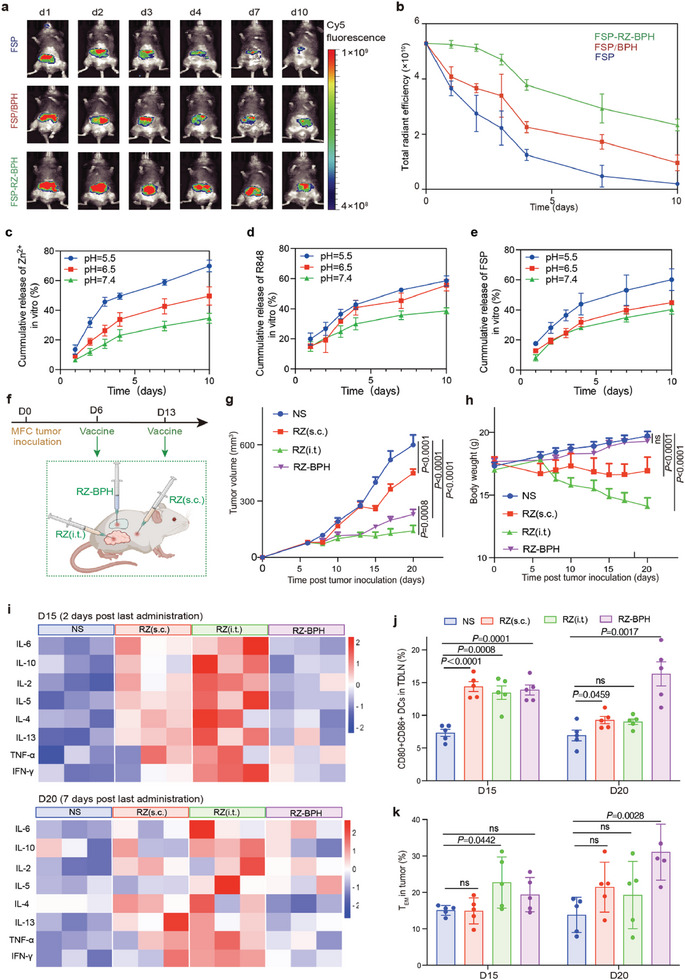
RZ‐BPH activates local immune responses with pH‐responsive drug release. a) Near‐infrared (NIR) imaging of 615 mice on D1, D2, D3, D4, D7and D10 after subcutaneous injection with FSP, FSP/BPH or FSP‐RZ‐BPH. FSPs were dyed with Cy5‐NHS. b) Total radiant efficiency in vivo of 615 mice after injection with FSP, FSP/BPH or FSP‐RZ‐BPH over time (n = 3). c) Cumulative release of Zn^2+^ from FSP‐RZ‐BPH in different pH in vitro (n = 3). d) Cumulative release of R848 from FSP‐RZ‐BPH in different pH in vitro (n = 3). e) Cumulative release of FSP from FSP‐RZ‐BPH in different pH in vitro (n = 3). f) Schematic diagram of different administrations of RZ in MFC tumor suppression experiment. g) Average tumor‐growth curves of 615 mice bearing MFC gastric cancer with different treatments as indicated in 20 days (n = 5). h) Average body weight of mice in different groups in 20 days (n = 5). i) Levels of the eight inflammatory cytokines (IL‐5, IL‐13, IL‐2, IL‐6, IL‐10, IFN‐γ, TNF‐α, IL‐4) in the serum of mice in different groups detected on D15 (2 days post last administration) and D20 (7 days post last administration) (n = 3). j) Percentage of CD80^+^CD86^+^ DCs in tumor‐draining lymph nodes (TDLNs) in different groups detected on D15 and D20 (n = 5). k) Percentage of T_EM_ in tumor in different groups detected on D15 and D20 (n = 5). The error bars represented mean ± SEM. *P*‐values were calculated by two‐way ANOVA and Tukey post‐test and correction g,h) or two‐tailed unpaired Student's t‐tests j,k). ns represented p > 0.05.

The sustained release effect of hydrogels can prolong the half‐life of neoantigen vaccines, which usually require multiple subcutaneous administrations. We compared the subcutaneous half‐life of FSP in NS, BPH, and FSP‐RZ‐BPH, and found that both hydrogels could prolong the half‐life, especially FSP‐RZ‐BPH, which increased the subcutaneous retention of FSPs by 11.5‐fold in D10 (**Figure** **3**a,b). Considering the acidic environment of TME, we hypothesized that Zn^2+^ bound to ‐SH under alkaline conditions are more likely to be dissociated and released at sites close to the tumor, while remaining relatively stable at sites far from the tumor. We tested the release of three pharmaceutical ingredients, Zn^2+^, R848 and FSPs at different pH, and found that their release rates all accelerated as the pH decreased. The pH‐responsive release of Zn^2+^ was the most significant one, whose release rate increased from 34.6% (pH 7.4) to 49.7% (pH 6.5) and 69.8% (pH 5.5) within 10 days (Figure 3c–e). Notably, the protein degradation may also contribute to the pH responsiveness. Moreover, the release of FSPs in FSP‐RZ‐BPH demonstrated an accelerated rate in the presence of glutathione (GSH) (Figure S8, Supporting Information). The pH‐ and GSH‐responsive hydrogel facilitated sustained immune responses from FSPs and the dual adjuvants, minimizing adverse effects.

### 2.5 RZ‐BPH Stimulated Local Immune Response

A potential concern for immunotherapy is the presence of a cytokine release syndrome (CRS) or cytokine storm after treatment. It has been proved that intratumoral (i.t.) and subcutaneous (s.c.) injections rather than intravenous injections are better administrations to activate local immune responses and avoid systemic inflammatory responses.^[^ ^25^ ^]^ We compared the anti‐tumor effects and safety of RZ under different administrations (Figure 3f). Compared to the NS group, RZ (s.c.) had a very limited inhibitory effect on tumor growth, and this may be due to the rapid metabolism of RZ in vivo. Both RZ (i.t.) and peritumoral injection of RZ‐BPH significantly slowed down tumor growth, and RZ (i.t.) seemed to be more effective (Figure 3g). However, compared with the NS and RZ‐BPH groups, the average body weight of the mice in the RZ (s.c.) and RZ (i.t.) groups continued to decrease, especially in the RZ (i.t.) group, whose average body weight was only 71.4% of that in the NS group (Figure 3h). We detected 8 inflammatory factors in the serum of mice in each group. On D15, 2 days after the last administration, the levels of the 8 inflammatory factors in the RZ (s.c.) and RZ (i.t.) groups increased significantly compared to the NS group, and the systemic inflammatory responses were the most pronounced in RZ (i.t.), whereas it was milder in the RZ‐BPH group. On D20, 7 days after the last administration, the systemic inflammatory response RZ (s.c.) and RZ (i.t.) groups decreased, but was still more severe than that in the NS and RZ‐BPH groups (Figure 3i). In the TDLNs, all administrations of RZ activated DCs on D15, but on D20, the proportion of mDCs in the RZ (s.c.) and RZ (i.t.) groups decreased, while the RZ‐BPH group remained a high level of mDCs, increasing effector memory T cells (T_EM_, CD3^+^CD8^+^CD44^+^CD62L^–^) in the tumor (Figure 3j,k).

### 2.6 FSP‐RZ‐BPH Inhibited the Growth of MFC Tumors

**Figure 4:**
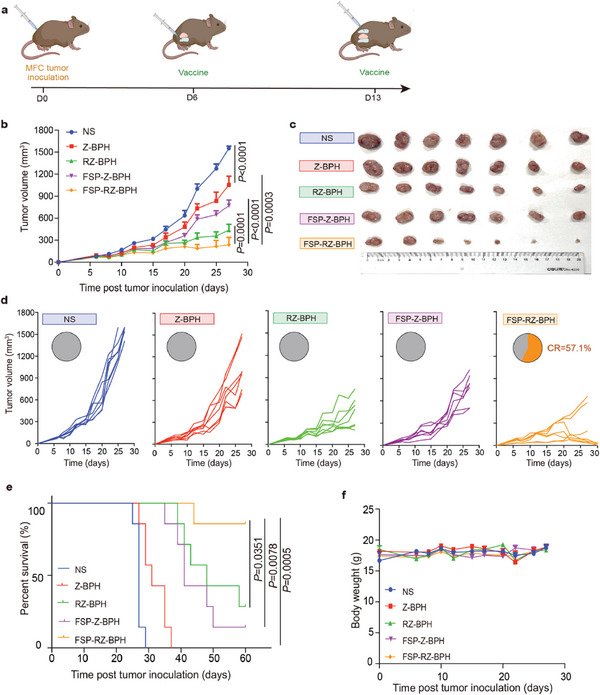
In vivo anti‐tumor effect of FSP‐RZ‐BPH. a) Schematic diagram of administration route of FSP‐RZ‐BPH in MFC tumor suppression experiment. b) Average tumor‐growth curves of 615 mice bearing MFC gastric cancer with different treatments as indicated in 27 days (n = 7). c) Photos of tumors harvested from mice in all groups on D23 (10 days after last administration) (n = 7). d) Tumor‐growth curves of each mouse in different groups (n = 7). CR represented complete response. e) Survival curves of 615 mice in different groups for 60 days (n = 7). f) Average body weight of 615 mice in different groups in 27 days (n = 7). The error bars represented mean ± SEM. *P*‐values were calculated by two‐way ANOVA and Tukey post‐test and correction b, f) or log‐rank (Mantel–Cox) test e). ns represented p > 0.05.

Neoantigen vaccines hold promise as personalized vaccine targets; however, our preliminary investigations have revealed their limited efficacy as single drug owing to the suppressive immune microenvironment prevalent in tumors.^[^ ^26^ ^]^ As elucidated in Figure S12, Supporting Information, the therapeutic efficacy of FSPs monotherapy is notably constrained. Analysis of the TME revealed that FSPs exerted minimal impact on the proportions of CD4^+^ and CD8^+^ T cells in TME. While a marginal reduction in Tregs and a modest increase in IFN‐γ^+^CD8^+^ T cells were discerned, statistical significance was not achieved. In this study, we aimed to enhance the effectiveness of neoantigen vaccines by refining delivery methodologies or integrating them with adjuvants. To verify the anti‐tumor effect of FSP‐RZ‐BPH, we established MFC subcutaneous tumors in 615 mice by inoculating 1 × 10^6^ MFC cells in the left lower abdomen of 615 mice (**Figure** **4**a). After 6 days, the tumors grow to ≈60–70 mm^3^. The mice in each group were randomly divided into 5 groups for treatment: NS, Z‐BPH, RZ‐BPH, FSP‐Z‐BPH, FSP‐RZ‐BPH. The tumor size recording was stopped when the tumor volume in the NS group was close to 1500 mm^3^ on day 27. The tumor growth curves showed that the four treatments all inhibited the tumor growth to varying degrees. The tumor volume of the Z‐BPH group was 1052.8 mm^3^ on day 27, while that of the RZ‐BPH group was only 437.2 mm^3^, whose tumor inhibitory effect was even better than FSP‐Z‐BPH, showing the great contribution of dual adjuvants (RZ) in regulating TME. The FSP‐RZ‐BPH group achieved 84.8% tumor inhibition rate, whose tumor volume was only 236.2 mm^3^ on day 27, and 57.1% (4/7) of the mice continued to shrink in tumor volume, and achieved CR in 60 days (Figure 4b–d). The survival curve of the mice showed that all the mice in the NS group died in 30 days, while the survival rate of the mice in the FSP‐RZ‐BPH group was 85.7% (6/7) in 60 days (Figure 4e). The local administration of hydrogel ensured the safety of the treatment, and there was no significant difference in the body weight of the mice in each group (Figure 4f).

### 2.7 FSP‐RZ‐BPH Activated Anti‐Tumor Response Through TDLNs

**Figure 5:**
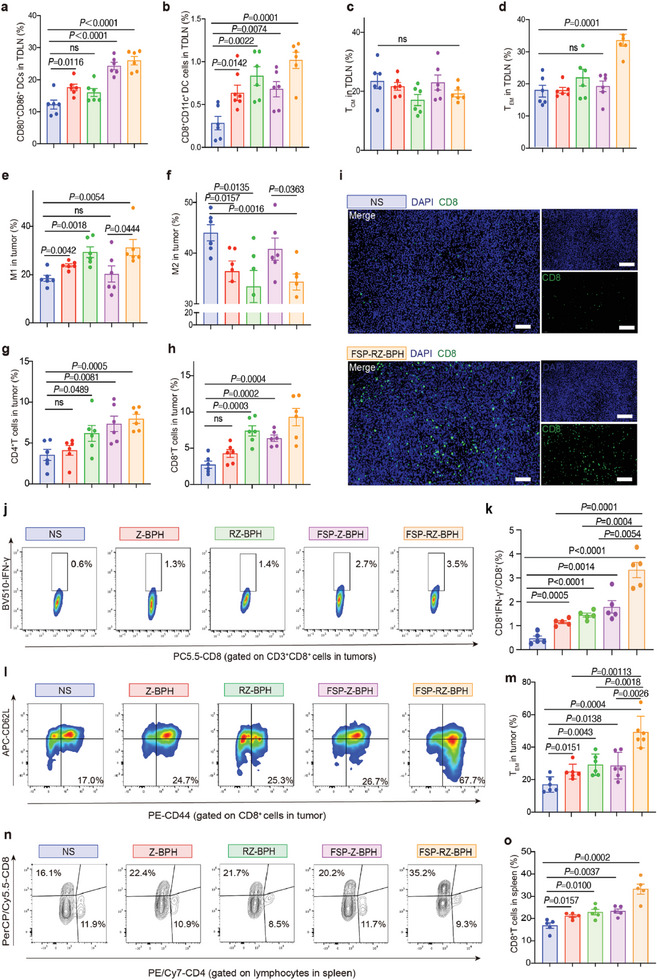
Immune response induced by FSP‐RZ‐BPH. a) Percentage of mature DCs (CD80^+^CD86^+^/CD11c^+^) in tumor‐draining lymph nodes (TDLNs) detected on D23 (n = 6). b) Percentage of CD8^+^CD11c^+^ DC (CD8^+^CD11c^+^/CD11c^+^) in TDLNs detected on D23 (n = 6). c) Percentage of central memory T cells (T_CM_, CD44^+^CD62L^+^/CD3^+^CD8^+^) in TDLNs detected on D23 (n = 6). d) Percentage of effector memory T cells (T_EM_, CD44^+^CD62L^−^/CD3^+^CD8^+^) in TDLNs detected on D23 (n = 6). e) Percentage of M1 tumor‐associated macrophage (M1‐TAM, CD86^+^/F4/80^+^CD11b^+^) detected on D23 (n = 6). f) Percentage of M2 tumor‐associated macrophage (M2‐TAM, CD206^+^/F4/80^+^CD11b^+^) detected on D23 (n = 6). g) Percentage of CD4^+^ T cells (CD4^+^/live cells) in tumor detected on D23 (n = 6). h) Percentage of CD8^+^ T cells (CD8^+^/live cells) in tumor detected on D23 (n = 6). i) Immunofluorescence staining of CD8^+^ cells in tumors of mice in NS and FSP‐RZ‐BPH groups (The scale bar is 100 µm in merge images and 200 µm in the other images). j) Representative flow cytometry images of CD8^+^IFN‐γ^+^ T cells in tumors in different groups detected on D23. k) Percentage of CD8^+^IFN‐γ^+^ T cells (CD8^+^IFN‐γ^+^/CD8^+^) in tumors in different groups detected on D23 (n = 5). l) Representative flow cytometry images of T_EM_ in tumors of mice in different groups detected on D23. m) Percentage of T_EM_ (CD44^+^CD62L^−^/CD3^+^CD8^+^) in tumors of mice in different groups detected on D23 (n = 6). n) Representative flow cytometry images of T cells in spleen of mice in different groups detected on D23. o) Percentage of T cells in spleen of mice in different groups detected on D23 (n = 6). The error bars represented mean ± SEM. *P*‐values were calculated by two‐tailed unpaired Student's t‐tests. ns represented p > 0.05.

After confirming the strong tumor‐suppressive effect of FSP‐RZ‐BPH, we detected the changes in immune‐related cells in TDLNs and tumors. In the TDLNs, the proportion of activated DCs in the FSP‐RZ‐BPH group increased from 12.3% in the NS group to 26.0% (**Figure** **5**a). Mature DCs (mDCs) were more efficient at delivering neoantigens to T cells, as demonstrated by a 3.6‐fold increase in the number of CD8^+^CD11c^+^ DCs (Figure 5b). T cells can be stimulated by mDCs loaded with neoantigens to differentiate into central memory T cells (T_CM_, CD3^+^CD8^+^CD44^+^CD62L^+^) and T_EM_. In the case of little difference in T_CM_, the proportion of T_EM_ in the FSP‐RZ‐BPH group reached 33.6%, which was significantly higher than the other four groups (Figure 5c,d). FSP‐RZ‐BPH elevated the proportion of mDCs and T_EM_ TDLNs, thereby ultimately augmenting the influx of immune cells in the tumor via peripheral circulation. We mainly detected the changes in macrophages and T cells in the tumor. The two groups containing the dual adjuvants (RZ‐BPH and FSP‐RZ‐BPH) had the most significant effect on macrophages, both of which could effectively convert the immunosuppressive M2 macrophages (F4/80^+^CD11b^+^CD206^+^) into immune‐promoting M1 macrophages (F4/80^+^CD11b^+^CD86^+^) (Figure 5e,f). An increase in tumor‐infiltrating T lymphocytes predicts a favorable immune response. The increase of tumor‐infiltrating T lymphocytes (TILs) predicts a favorable response to immunotherapy.^[^ ^27^ ^]^ FSP‐RZ‐BPH significantly increased the number of TILs, including CD4^+^ and CD8^+^ TILs (Figure 5g–i). In the CD4^+^ T cells, the proportion of regulatory T cells (Tregs) in the FSP‐RZ‐BPH group underwent a substantial reduction, reaching a mere 2.0%. This value markedly contrasts with the notably higher 16.1% observed in the NS group (Figure S10, Supporting Information). Compared to the NS group, the number of CD8^+^ TILs in the FSP‐RZ‐BPH group increased by 3.4 fold, specifically, CD8^+^ IFN‐γ^+^ T cells in the FSP‐RZ‐BPH group were 9.8 fold more abundant than those in the NS group (Figure 5j,k). CD8^+^ T_EM_ can secrete γ‐IFN or granzyme B in a very short time to kill tumor cells after exposure to pre‐inoculated neoantigens, whose proportion increased in all treatment groups, and the FSP‐RZ‐BPH had the highest proportion, reaching 49.3% (Figure 5l,m). In addition, we observed an increase in the proportion of CD8^+^T cells in splenocytes, which indicated the long‐term immune effect of FSP‐RZ‐BPH treatment and its potential as a preventive vaccine (Figure 5n,o).

### 2.8 FSP‐RZ‐BPH Activated Innate and Adaptive Immunity

**Figure 6:**
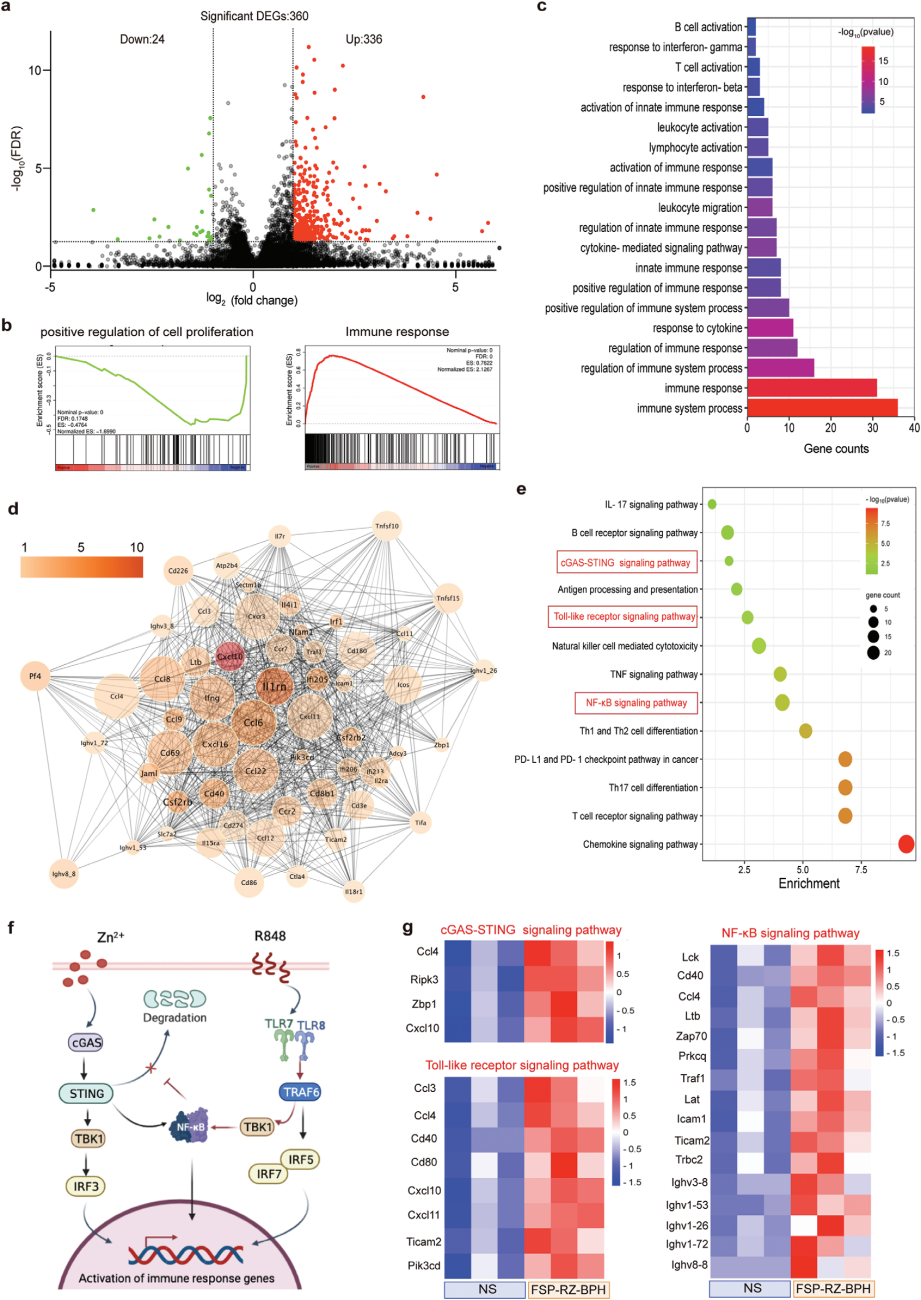
RNA‐seq analysis of tumors after FSP‐RZ‐BPH administration. a) Volcano map of differentially expressed genes (DEGs) between the NS and FSP‐RZ‐BPH groups (n = 3). b) Gene Set Enrichment Analysis (GSEA) analysis of cell proliferation and immune response (n = 3). c) Gene Ontology (GO) analysis of upregulated genes (n = 3). d) GO network analysis of significantly upregulated genes in tumors of mice in FSP‐RZ‐BPH compared with the NS‐treated mice. Color scales represented the values of log2‐transformed fold changes and the circle size represented gene enrichment (n = 3). e) Kyoto Encyclopedia of Genes and Genomes (KEGG) enrichment analysis (n = 3). f) Schematic diagram to demonstrate the mechanism by which R848 and Zn^2+^ exert synergistic pro‐inflammatory effects as TLR7/8 agonists and cGAS‐STING agonists, respectively. g) Differential genes related to cGAS‐STING, Toll‐like receptor and NF‐κB signaling pathways. The high‐expression genes and low‐expression genes were clustered by log 10 (FPKM+1) (n = 3).

The powerful anti‐tumor effect of FSP‐RZ‐BPH came from the innate immunity activated by dual adjuvants (RZ) enhancing the FSPs‐specific adaptive immunity. To confirm this underlying mechanism, we performed RNA sequencing (RNA‐seq)‐based transcriptome analysis of treated tumors. As shown in **Figure** **6**a, compared with the NS group, 336 genes were significantly up‐regulated and 24 genes were down‐regulated after FSP‐RZ‐BPH treatment. Gene set enrichment analysis (GSEA) showed that down‐regulated genes were mainly related to “positive regulation of cell proliferation”, while “immune response” related genes showed an upward trend (Figure 6b). The results of gene ontology (GO) analysis were similar, and the up‐regulated genes were mostly concentrated in immune response, innate immune response and lymphocyte activation (Figure 6c). Among these up‐regulated genes, IL1rn, IFNg and chemokines such as CXCL10, CXCL16, CCL6, CCL8 played key roles and are the link between innate immunity and adaptive immunity (Figure 6d). Kyoto encyclopedia of genes and genomes (KEGG) analysis showed that the activation of adaptive immunity represented by T cells was accompanied by the activation of innate immunity represented by NK cells. Among them, TLRs signaling pathway and cGAS‐STING signaling pathway played key roles (Figure 6e). R848 and Zn^2+^ are the agonists of TLRs and cGAS‐STING, respectively. It had been proved the activation of TLRs signaling pathway could enhance the effect of NF‐κB molecules to inhibit the degradation of STING molecules,^[^ ^18^ ^]^ thereby playing a synergistic role with cGAS‐STING agonists in promoting immunity (Figure 6f). After FSP‐RZ‐BPH treatment, TLRs signaling pathway, cGAS‐STING signaling pathway and NF‐κB signaling pathway related genes were significantly up‐regulated compared to the NS group (Figure 6g), and this proved the possible mechanism for R848 and Zn^2+^ to play a synergistic immune‐promoting effect.

### 2.9 FSP‐RZ‐BPH Generated Neoantigen‐Specific Immune Memory Responses

**Figure 7:**
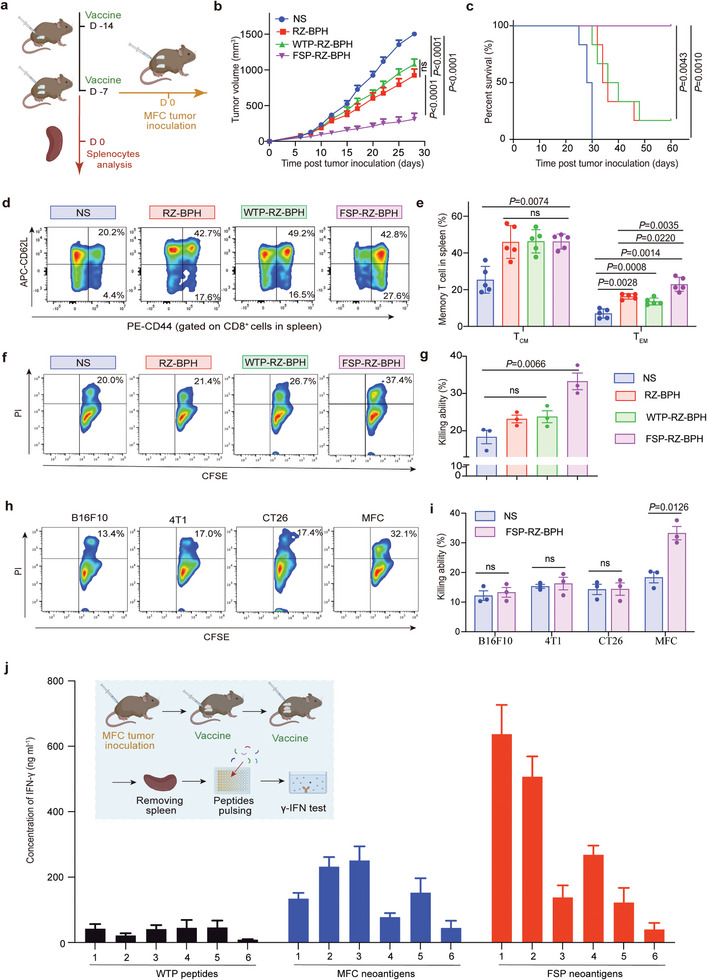
FSP‐RZ‐BPH induced MFC‐specific immune memory responses. a) Schematic diagram to show the administration route of pre‐immunized FSP‐RZ‐BPH in MFC tumor prevention experiment and splenocytes analysis. b) Average tumor‐growth curves of 615 mice bearing MFC gastric cancer with different treatments as indicated in 28 days (n = 6). c) Survival curves of 615 mice in different groups for 60 days (n = 6). d) Representative flow cytometry images of T_CM_ and T_EM_ in spleen of mice in different groups detected on D0. e) Percentage of T_CM_ and T_EM_ in spleen of mice in different groups detected on D0 detected on D23 (n = 5). f) Representative flow cytometry images of CFSE^+^PI^+^ MFC tumor cells after co‐incubation with splenocytes of mice after different treatments. g) Killing ability of splenocytes to MFC tumors after different treatments (n = 3). h) Representative flow cytometry images of CFSE^+^PI^+^ cells of four different tumor cells (B16F10, 4T1, CT26 and MFC cells) after co‐incubation with splenocytes of mice after FSP‐RZ‐BPH treatment. i) Killing ability of splenocytes to different tumor cells after NS or FSP‐RZ‐BPH treatment (n = 3). j) Concentration of IFN‐γ secreted by splenocytes of 615 mice stimulated by WTP peptides (abscissa coordinates 1–6 represented WTP‐1 to WTP‐6), MFC neoantigens (abscissa coordinates 1–6 represented MFC‐1 to MFC‐6) or FSP neoantigens (abscissa coordinates 1–6 represented FSP‐1 to FSP‐6). Before testing, the mice were inoculated with MFC tumor cells and treated with FSP‐RZ‐BPH twice as the schematic diagram showed (n = 3). The error bars represented mean ± SEM. *P*‐values were calculated by two‐way ANOVA and Tukey post‐test and correction b), log‐rank (Mantel–Cox) test c) or two‐tailed unpaired Student's t‐tests e,g,i). ns represented p > 0.05.

Activation of innate immunity can greatly promote neoantigen‐specific adaptive immunity. In order to verify the specific anti‐tumor immune response generated by FSP‐RZ‐BPH, we established a MFC prevention model in 615 mice. 615 mice were pre‐immunized twice with FSP‐RZ‐BPH before inoculated with MFC cells (**Figure** **7**a). It could be found that the tumor growth rate in WTP‐RZ‐BPH group was slightly slower than the NS group and close to the RZ‐BPH group, but significantly faster than the FSP‐RZ‐BPH group, indicating that FSP pre‐inoculation was key in the anti‐tumor immune memory, thereby slowing tumor growth and prolonging the survival of MFC‐bearing mice (Figure 7b,c). Spleen is the major storage site for immune memory cells, and we performed immunoassays on mouse spleen tissues 7 days after the last pre‐inoculations. The increase of T_CM_ cells in the three treatment groups was similar, but the difference in T_EM_ was huge. The T_EM_ in NS group was only 7.0%, while the T_EM_ of the FSP‐RZ‐BPH group was significantly increased to 22.9%, which was also significantly higher than that in RZ‐BPH group (16.3%) and WTP‐RZ‐BPH group (13.6%) (Figure 7d,e). In order to compare the specificity of splenic memory T cells, we tested the killing ability of splenocytes induced by different administration methods on different tumor cells. First, we compared the killing effect of splenocytes on MFC induced by the above four different administration methods. Similar to the trend of tumor growth, the killing ability of WTP‐RZ‐BPH group and RZ‐BPH group was similar, slightly higher than that of NS group (18.3%), while the killing ability of FSP‐RZ‐BPH group to splenocytes was significantly improved to 33.3% (Figure 7f,g). Afterward, FSP‐RZ‐BPH‐induced splenocytes were used to kill four different tumor cells: B16F10, 4T1, CT26 and MFC cells. The killing effect of 615 mouse splenocytes on MFC in the untreated group (NS) was slightly higher than that of the other three types of cells, but there was no statistical difference. After FSP‐RZ‐BPH pre‐immunization, the killing effect of 615 mouse splenocytes on MFC was increased to 33.3%, while the splenocytes still exhibited low killing ability on the other three tumor cells (Figure 7h,i). The above results indicated that FSP‐RZ‐BPH generated an immune memory response specific to MFC tumors, and FSPs were key to the specificity.

### 2.10 FSP‐RZ‐BPH Produced Epitope Spreading Effect

In the prevention model, we demonstrated that pre‐inoculation of FSP‐RZ‐BPH generated MFC‐specific T cells in the spleen. To further verify whether splenocytes have an immune memory response to neoantigens on MFC cells after FSP‐RZ‐BPH treatment, in the MFC subcutaneous tumor suppression model, we obtained splenocytes of mice after FSP‐RZ‐BPH treatment to test its response to WTPs, FSPs and MFCs. As expected, the obtained splenocytes had a strong memory response to FSPs and secreted a large amount of IFN‐γ after stimulation by FSPs. Interestingly, concentrations of IFN‐γ stimulated by MFCs were also elevated compared to WTPs, although there was no prior vaccination against MFCs (Figure 7j). These results suggest that NRTs generated by FSP‐RZ‐BPH treatment produced memory immune responses not only to administrated neoantigens but also to the other non‐immunized neoantigens on MFC cells. This epitope spreading effect, also referred to as the bystander effect, favored the continued evolution of specific anti‐tumor immune responses against immune tolerance under selection stress.

### 2.11 Biosafety Assessment

In the construction of FSP‐RZ‐BPH, we paid special attention to potential security issues. Subcutaneous or intratumoral injection of the dual adjuvants RZ caused a severe systemic inflammatory response, making the mice to lose weight abnormally, while FSP‐RZ‐BPH only caused a slight systemic inflammatory response, and the mouse body weight remained at a normal level (Figure 3h,i). When applied to the treatment of subcutaneous MFC tumor in 615 mice, compared with the NS group, the mice in the other four groups did not have abnormal body weight fluctuations (Figure 4f). We also detected the liver and kidney functions of the mice in each group. It could be seen that the levels of aspartate aminotransferase (AST), alanine aminotransferase (ALT), alkaline phosphatase (ALP), blood urine nitrogen (BUN), creatinine (CREA) were basically consistent and within the normal range (Figure S14, Supporting Information). In addition, the H&E staining images of major organs (heart, liver, spleen, lung, kidney) showed there were no obvious damage in each group (Figure S15, Supporting Information). These results indicated that FSP‐RZ‐BPH exerted significant local immune activation, induced only low‐grade systemic inflammation, and did not significantly impair liver and kidney functions in mice. Overall, the thermosensitive bi‐adjuvant hydrogel was examined with good biological safety.

## 3 Discussion

Neoantigen vaccine is a breakthrough strategy to induce highly efficient individualized anti‐tumor immune responses, but its efficacy varies significantly among different tumor types and populations. Several clinical trials showed that neoantigen peptide vaccines achieved good efficacy in melanoma and lung cancer, and the vast majority of neoantigens in these clinical trials are derived by SNVs, which may be due to their easier screening, identification, and mutation abundance.^[^ ^6^, ^7^ ^]^ However, the efficacy of SNVs‐derived neoantigens peptides in tumors with low mutation burden is not satisfactory. To develop mRNA‐based vaccines or engineering peptides vaccines and combined ICIs can enhance the immune response to neoantigens.^[^ ^6^, ^28^ ^]^ In previous work, we have identified SNVs‐derived neoantigen peptides MFCs for 615 mice, and enhanced its anti‐tumor effect by improving its lymph node targeting through nano‐modification and adjuvant co‐delivery.^[^ ^21^ ^]^ Recently, multiple studies have confirmed the efficacy and safety of highly immunogenic frameshift mutant neoantigen vaccines generated by DNA mismatch repair.^[^ ^5^, ^10^ ^]^ On this basis, we identified frameshift peptides neoantigens FSPs on MFC tumors and compared their immunogenicity with SNVs‐derived neoantigens MFCs. In the co‐incubation of T cells in vitro, the IFN‐γ produced by FSPs stimulation was tens or even hundreds of folds higher than that of the SNVs‐derived MFCs. This suggested targeting frameshift mutations was a better choice to develop neoantigen vaccines for tumors with low mutation burden.

After inoculation in vivo, neoantigens need to be cross‐presented by DCs to communicate with T cells, and its curative effect is affected by TME. There was a study using dual adjuvants CPG (TLR9 agonists) and R848 (TLR7/8 agonists) to improve the efficacy of neoantigen vaccine.^[^ ^29^ ^]^ In this study, we chose R848 and Zn^2+^ as dual adjuvants to construct a thermosensitive neoantigen‐adjuvant hydrogel. In comparison to previously reported cancer vaccines incorporating antigens, adjuvants, and hydrogel carriers, the innovations in this study are evident in several key aspects: First, we deviate from the use of SNV‐derived neoantigens, opting instead for the identification and verification of de novo MFC‐specific frameshift mutation neoantigens. This marks the inaugural report of frameshift MFC neoantigens characterized by heightened immunogenicity. Of particular significance is the multifaceted role of Zn^2+^ in the hydrogel vaccine, where it not only serves as a STING agonist but also harmoniously integrates with various components to enhance the overall biological functions, as well as the chemical and physical properties of the vaccine: 1. Zn^2+^ contributes to the reduction of the gelation time of BSA‐PEG without a concomitant increase in degradation time. Additionally, it imparts pH responsiveness to the hydrogel, thereby facilitating sustained drug release in the TME; 2. The combination of Zn^2+^ and R848 is underscored by its robust and extensive synergistic pro‐inflammatory effect. This finding holds practical implications for the clinical co‐administration of STING agonists and TLR agonists; 3. The formation of S─Zn bonds establishes a connection between FSPs and the BSA skeleton, enhancing the sustained‐release properties of the hydrogel. These distinctive characteristics render Zn^2+^ indispensable, differentiating it from other STING agonists and metal ions (Mn^2+^, Mg^2+^, etc.).

Immune tolerance is another important factor that limits the efficacy of immunotherapy. A typical example is the exhaustion of T cells associated with the upregulation of PD‐1 expression after immunotherapy. For personal neoantigen vaccine, it has been found that under the selection pressure of NRTs, the tumor antigens expression will undergo Darwinian evolution, that is, the targeted neoantigens will be down‐regulated, while the load of untargeted neoantigens will increase, and even produce new genomic mutations. These epitope changes will induce NRTs to lose specific cytotoxicity against tumor cells.^[^ ^30^ ^]^ We are excited to find that splenocytes of mice treated with FSP‐RZ‐BPH had memory responses not only to FSPs, but also to uninoculated neoantigens. This is due to the direct effect of RZ‐induced ROS killing tumors to release sufficient number of neoantigens and the anti‐tumor immune cycle formed by long‐lasting immune‐stimulating effect of the hydrogel. This epitope spreading effect usually predicts prolongation in progression‐free and overall survival,^[^ ^31^ ^]^ which indicates FSP‐RZ‐BPH administration is able to reduce self‐tolerance and produce long‐term MFC‐specific immune responses.

In summary, FSP‐RZ‐BPH stands out as a novel hydrogel, featuring the first‐ever reported frameshift neoantigens, potent dual adjuvants, and TME‐responsive sustained‐release properties. There were some limitations and future plans in our work. When verifying the broad‐spectrum activation of RZ, a comprehensive understanding of the underlying mechanisms is crucial. To elucidate this, we have executed RNA‐sequencing, depicted in Figure 6g, confirming the upregulation of genes associated with TLRs, STING, and NF‐κB signaling pathways. However, the inclusion of key proteins integral to these pathways are also important to enhance the robustness of our proposed mechanistic framework. In subsequent investigations, we are steadfast in our commitment to undertaking western blot experiments, aiming to corroborate alterations in the expression levels of critical proteins such as STING, TBK1, IRF3, TRAF6, and others, under the influence of FSP‐RZ‐BPH. Besides, immune pressure will increase the expression of inhibitory molecules such as PD‐1 and TIM‐3 and cause the exhaustion of T cells. Combining FSP‐RZ‐BPH with anti‐PD‐1 therapy is worthy of further exploration to better inhibit tumor growth. In addition, in view of the epitope spreading effect, further exploring the role of FSP‐RZ‐BPH in models of abdominal dissemination or lung metastasis of gastric cancer could provide stronger evidence for its clinical application.

## 4 Conclusion

In this study, we successfully created a thermosensitive bi‐adjuvant hydrogel simultaneously targeting innate and FSPs‐specific immunity. FSPs are the first reported MFC frameshift neoantigens to be more immunogenic than SNVs‐derived MFC neoantigens. Zn^2+^ played an important link role in the formation of FSP‐RZ‐BPH: direct ROS activation, synergistic pro‐inflammatory effect with R848, structurally linking FSPs and improving the hydrogel performance. The engineered hydrogel could release FSPs and adjuvants in a pH‐responsive manner to reduce the toxic side effects and effectively improve drug utilization, thus generating a large number of activated DCs in TDLNs and increasing the number of tumor‐infiltrating T_EM_ by 2.9 folds. These T_EM_ displayed epitope spreading effects to avoid immune tolerance, and had durable memory responses to MFC tumors. In the subcutaneous MFC tumor suppression model, FSP‐RZ‐BPH achieved 84.8% tumor inhibition rate and prolonged the survival of tumor‐bearing mice with a 57.1% complete response rate within 60 days. Besides, FSP‐RZ‐BPH can also be used as a preventive vaccine. Altogether, our work identified frameshift MFC neoantigens for the first time and demonstrates an effective strategy to enhance its anti‐tumor efficacy in an injectable thermosensitive hydrogel.

## 5 Experimental Section

### Materials, Cell Lines and Mice

R848 was purchased from MedChemExpress (USA). ZnCl_2_ was purchased from Aladdin Industrial Corporation (Aladdin, Shanghai, China). Roswell Park Memorial Institute (RPMI) 1640 medium, fetal bovine serum (FBS), collagenase IV, and penicillin‐streptomycin (P/S) were purchased from Gibco.

MFC gastric cancer cells, 4T1 breast cancer cells, B16F10 melanoma cells, MC38 colorectal cancer cells, and RAW264.7 cells were obtained from the cell bank of Shanghai Institute of Biochemical Cell Biology and cultured with RPMI 1640 containing 10% FBS and 1% P/S cultured at 37 °C, 5% CO_2_. All cell lines were tested for mycoplasma and only mycoplasma‐free cells were used. 615 and C57BL/6 female mice aged 5–6 weeks were purchased from Shanghai Sippr‐BK laboratory animal Co. Ltd (Shanghai, China) and bred at the Specific Pathogen Free (SPF) Experimental Animal Center of Affiliated Nanjing Drum Tower Hospital of Nanjing University Medical School. All animal experiment protocols were approved by the Experimental Animal Care and Use Committee of Affiliated Nanjing Drum Tower Hospital of Nanjing University Medical School (2023AE01016).

### FSPs Prediction and Validation

Tumor tissue specimens and blood samples were collected from MFC tumor‐bearing 615 mice and subjected to WES (Shanghai Biotecan Pharmaceuticals Co., Ltd, Shanghai, China). The identified non‐synonymous mutations were classified according to frequency, and the PubMed nucleotide tool was used to identify amino acids of the wild‐type protein. The mean depth was 500X for each sample after PCR duplicates removal. The reads were paired and 150 bp length reads were aligned by BWA mem, followed by PCR duplicates removal. The raw calls of SNVs and short indel were further selected as follows. A minimum of 5 reads was required to support alternative calling. Variants with read depths less than 30x with strand bias larger than 10% or VAF < 0.5% were removed. Q30 > = 75% for each sample; lianti r116(sSNV and indel identification) was used for SNV/InDels. The top 200 high‐incidence(>5%)of true somatic mutations of coding microsatellites (cMS) was extracted whose sequence on the reference genome was at least 8 consecutive identical bases. SYFPEITHI (http://www.syfpeithi.de/0‐home.htm) and NetMHCpan‐4.1 (https://services.healthtech.dtu.dk/service.php?NetMHCpan‐4.1) were used for H‐2K^k^ (615 mouse HLA type)binding peptide prediction, and 8–11 mer mutant peptides with a binding affinity half‐maximum inhibitory concentration < 200 nM were selected.

The selected 6 FSPs were synthesized by Bankpeptide Biotechnology (Hefei, China). To verify whether the screened FSPs were immunogenic, splenocytes were obtained from 615 mice for peptides pulse. 1 × 10^5^ splenocytes in a 96‐well plate were stimulated with 10 µg ml^−1^ of each peptide on D0, and stimulated again on D3. Remove all supernatants on D6, and add the peptides to the corresponding cells in 96‐well plate to co‐culture for 24 h. Finally, the supernatant was collected and detected with BD Cytometric Bead Array (CBA) Mouse IFN‐γ Enhanced Sensitivity Flex Set and BD CBA Mouse Enhanced Sensitivity Master Buffer Kit.

### The Pro‐Inflammatory Effect of R848 and Zn^2+^

Mainly flow cytometry was used to test the effects of R848 and Zn^2+^ on DCs, macrophages, T cells, and MFC tumor cells. The concentration of R848 used in all cell experiments was 3 ug ml^−1^, and the concentration of Zn^2+^ was 50 µM. For DCs, APC anti‐mouse CD80 antibody and PE anti‐mouse CD86 antibody were used to detect the activation of DCs, while PE anti‐mouse H‐2K^K^ antibody and APC anti‐mouse IA/IE antibody were used to detect the expression of MHC I and MHC II molecules on DCs. For T cells, PE/Cy7 anti‐mouse CD4 antibody, APC anti‐mouse CD8 antibody were used for the classification of T cells, and PerCP/Cy5.5 anti‐mouse CD69 antibody, FITC anti‐mouse CD25 antibody were used as markers of early and late activation of T cells. For macrophages, FITC anti‐mouse CD11b antibody and PE/Cy7 anti‐mouse F4/80 antibody were used to identify macrophages, and PE anti‐mouse CD86 antibody, APC anti‐mouse CD206 antibody were used as markers of M1 and M2 macrophages, respectively. In this study, all the monoclonal antibodies were purchased from Biolegend (USA) and all the flow data were acquired with a BeckMan CytoFlex (USA) and analyzed by FlowJo software (USA).

The steps of the cell experiment have been described in the previous literature.^[^ ^32^ ^]^ Briefly, for BMDCs, bone marrow mesenchymal stem cells were obtained from the femur and tibia of C57BL/6 mice aged 5–6 weeks, and the harvested cells were cultured with 10% FBS and 1% P/S in RPMI 1640 medium. Add 20 ng ml^−1^ rmGM‐CSF (Xiamen Aituo Biotechnology Co., Ltd., China) and 10 ng ml^−1^ rmIL‐4 (Pepro Tech, USA) to induce the cells to differentiate into DCs. During the culture period, three‐quarters of the medium was changed every 2–3 days, and all supernatants were discarded on D8 to collect all cells. The harvested immature DCs were resuspended in complete medium without cytokines, plated in a 96‐well plate at a concentration of 1 × 10^6^ cells per well, and co‐cultured with the indicated drugs for 24–48 h. Finally, all cells were collected by centrifugation at 500 g for 5 min, and then incubated with 1 µl of the corresponding monoclonal antibody for 30 min before flow cytometry testing.

In order to verify the ability of BMDCs to present FSPs, after the immature DC was obtained according to the above method, FSPs were added for co‐incubation. 4 h later, the BMDCs were treated with NS or RZ for 24–48 h. Remove the supernatant and washed the cells with PBS for 2–3 times. A confocal laser scanning microscopy (CLSM, FV1000, Olympus, Japan) was used to observe and take the images.

615 mouse‐derived splenocytes were harvested for T cells testing. Red blood cells in the spleen were lysed with lysis buffer (Invitrogen eBioscience) for 5 min, the remaining cells were collected and added to 96‐well plates at 1 × 10^6^ cells ml^−1^ with R848/Zn in 100 µl AIMV medium containing 10% FBS. After co‐incubating with R848 and Zn for 24–48 h, all cells were collected by centrifugation at 500 g for 5 minutes, and then incubated with 1 µL of the corresponding monoclonal antibody for 30 min before flow cytometry testing.

RAW264.7 cells were used for macrophages testing. RAW264.7 cells in the logarithmic growth phase was re‐plated at 96‐well plates at 1 × 10^5^ cells ml^−1^ in 100 µl 1640 medium containing 10% FBS, and R848 or Zn^2+^ were added for co‐incubating for 24–48 h. All cells were collected by centrifugation at 500 g for 5 min, and then incubated with 1 µl of the corresponding monoclonal antibody for 30 min before flow cytometry testing.

### Construction and Characterization of FSP‐RZ‐BPH

The formation of FSP‐RZ‐BPH involved three main processes: a. Formation of BSA‐PEG: Dissolve 1 g BSA in 40 ml PEG200, then add 1 ml 98% concentrated sulfuric acid dropwise in a fume hood, and stir overnight at room temperature. After that, add 600 ml of ethanol‐ether mixture (1:1, v/v) to the stirred liquid to obtain a precipitate, and transfer the precipitate to a 12 KDa dialysis bag with pure water for dialysis overnight. The dialysis liquid was fully lyophilized to obtain BSA ‐PEG powder; b. Formation of R848‐BSA‐PEG: Dissolve 160 mg BSA‐PEG lyophilizate, 3.8 mg EDC, 2.3 mg NHS in 5 ml double deionized water (dd‐water) and stir at room temperature for 15 min; then add 7 µl 2‐mercaptoethanol and stir at room temperature for 15 min. Add 500 µg R848 and adjust the pH of the solution to 7.2–7.5 by PBS, then the solution was placed at room temperature for 2–4 h and lyophilize after overnight dialysis in a 0.5 KDa dialysis bag to obtain BSA‐PEG‐R848 lyophilizate; c. Formation of FSP‐RZ‐BPH: Dissolve the FSPs (100 µg peptide^−1^),160 mg R848‐BSA‐PEG lyophilizate and 6.8 mg Zn^2+^ in 700 µl NS, then add 300 µl of sodium hydroxide solution to adjust the pH to 10.0. Then stir the solution overnight and lyophilize after overnight dialysis in a 0.5 KDa dialysis bag to obtain FSP‐RZ‐BSA‐PEG lyophilizate. To form the FSP‐RZ‐BPH, add 100 mg FSP‐RZ‐BSA‐PEG to 1 ml NS, and the hydrogel can form at 37 °C.

High performance liquid chromatography (HPLC, Agilent, USA) was used to detect the formation of FSP‐RZ‐BPH. The HPLC method was set as below: wavelength: 220 nm, column temperature: 30 °C, injection quantity: 20 µl, velocity of flow: 1 ml/min, mobile phase ratio: 1/1000 TFA Acetonitrile: 1/1000 TFA water = 10:90–65:35 17 min. Afterward, the morphology and elements of the hydrogel were examined. The aqueous solutions of BSA‐PEG and FSP‐RZ‐BSA‐PEG were placed in water baths at different temperatures for a period of time and then taken out. When the container was inverted and no liquid flowed down for at least 30 s, the hydrogels were formed. Afterward, the hydrogels were lyophilized and coated with a thin layer of gold, the morphology of the hydrogel was observed using a scanning electron microscope (SEM, HITACHI, Japan) at an accelerating voltage of 15 kV. On this basis, SEM with energy‐dispersive X‐ray spectroscopy (SEM‐EDS) was used to detect elemental mapping in FSP‐RZ‐BPH.

### Near‐Infrared Imaging of Mice

The subcutaneous half‐life of FSPs in different carriers was observed using an animal NIR imaging system. Before shooting, all FSPs were linked by Cy5‐NHS (Tocris Bioscience, Australia), then FSP‐RZ‐BPH was constructed according to the aforementioned process and injected subcutaneously in the abdomen of 615 mice. 615 mice were anesthetized and scanned at indicated time points (D0, 1, 2, 3, 4, 7, 10) using a CRi Maestro In Vivo Imaging System (Cambridge Research & Instrumentation, Massachusetts, USA) (n = 3).

### Drug Release of FSP‐RZ‐BPH

Add 2 ml of FSP‐RZ‐BP aqueous solution to the upper layer of a 6‐well transwell plate at 37 °C until the hydrogel formed. Among them, FSP1 was used as a model neoantigen peptide to represent FSPs. Then add 2 ml of aqueous solution with different pH (5.5, 6.5 and 7.4) to the lower layer of transwell. Put the transwell in a constant temperature box at 37 °C. Collect all the aqueous solution in the lower layer at the indicated time point and add new aqueous solution. Inductively coupled plasma optical emission spectrometer (ICP‐OES, USA) was used to detect the content of Zn^2+^ in the collected solution. HPLC was used to detect the content of R848 and FSP1 and the detection condition was described as above.

### Detection of Inflammatory Factors

The blood was collected from the mice by enucleation, and centrifuged to separate the serum. The concentration of the eight factors (IL‐5, IL‐13, IL‐2, IL‐6, IL‐10, IFN‐γ, TNF‐α, IL‐4) in the serum was detected by LEGENDplex MU Th1/Th2 Panel (8‐plex) w/VbP V03 (Biolegend, USA).

### In Vivo Anti‐Tumor Effect of FSP‐RZ‐BPH

In the MFC subcutaneous tumor suppression model, 1 × 10^6^ MFC cells were injected subcutaneously into the left lower abdomen of each 615 mice aged 5–6 weeks in 100 µl PBS solution. After 6 days, the tumor volume of the mice reached ≈75 mm^3^. Then, the mice were randomly divided into 5 different groups for treatment on D6 (n = 7): (1) NS, (2) Z‐BPH, (3) RZ‐BPH, (4) FSP‐Z‐BPH, (5) FSP‐RZ‐BPH. The dosage of the drug used for each mouse was as follows: 5 ug R848, 10 mm Zn, 100 ug FSP/peptide, which were dissolved in NS or BSA‐PEG solution to a final volume of 100 µl per dose. All mice received a second treatment on D13. The tumor volume and body weight of the mice were recorded every 2–3 days for 30 days. Tumor volume was measured by the following equation: V = (width)^2^ × length/2. The survival of the mice was observed for 60 days, and the mice were recorded as dead when the mice died or the tumor volume reached 1500 mm^3^.

In the MFC subcutaneous tumor prevention model, 6‐week‐old 615 mice were randomly divided into 4 groups on D‐14 (n = 6): (1) NS, (2) RZ‐BPH, (3) WTP‐RZ‐BPH, (4) FSP‐RZ‐BPH. After two vaccine pre‐immunizations on D‐14 and D‐7, 1 × 10^6^ MFC cells in 100 µl PBS were subcutaneously injected into the left lower abdomen of each 615 mice on D0. Similar to the tumor inhibition model, the tumor growth curve in 30 days and the survival curve in 60 days of the mice were recorded.

### Immune Response Induced by FSP‐RZ‐BPH

In MFC subcutaneous tumor suppression model, mice in each group were sacrificed on D23, 10 days after the last treatment, and left inguinal lymph nodes and tumor tissues were taken out (n = 6). The minced tumor tissue was digested with collagenase type IV (1 mg ml^−1^, Sigma) at 37 °C for 2 h under gentle agitation, and then a single‐cell suspension of inguinal lymph nodes was prepared by mechanical grinding. All cell samples were suspended in NS and stained with corresponding flow cytometry antibodies for 30 min at 4 °C in the dark. After washing 2 times, flow cytometry analysis was performed using a BeckMan CytoFlex. FITC anti‐mouse CD11c antibody, PE‐anti‐mouse CD86 antibody and APC‐anti‐mouse CD80 mAb were used to test the activation of DCs. FITC anti‐mouse CD11c antibody and APC anti‐mouse CD8 antibody were used to analyze the proportion of CD8^+^ DC in lymph nodes. PE/Cy7 anti‐mouse CD4 antibody, PE anti‐mouse CD44 antibody, PerCP/Cy5.5 anti‐mouse CD8 antibody, APC anti‐mouse CD62L antibody were used to identify T_CM_ and T_EM_ in lymph nodes and tumors. FITC anti‐mouse CD11b antibody, PE/Cy7 anti‐mouse F4/80 antibody were used to identify macrophages, and PE anti‐mouse CD86 antibody, APC anti‐mouse CD206 antibody were used as markers of M1 and M2 macrophages.

In the MFC subcutaneous tumor prevention model, 7 days after the last pre‐immunizations (D0), the mice in the 4 groups were sacrificed to collect the spleens (n = 5). The single‐cell suspension of splenocytes was prepared by mechanical grinding method and treated with lysis buffer for 5 min to remove red blood cells. All samples were suspended in NS and stained with corresponding flow cytometry antibodies for 30 min at 4 °C in the dark. After washing 2 times, flow cytometry analysis was performed. FITC anti‐mouse CD3 antibody, PE anti‐mouse CD44 antibody, PerCP/Cy5.5 anti‐mouse CD8 antibody, APC anti‐mouse CD62L antibody was used to identify the proportion of T_CM_ and T_EM_.

### Mouse Tumor RNA Sequencing and Gene Expression Analysis

Following the completion of the treatment regimen, 615 mice were euthanized two days post the last treatment. Tumors were promptly excised and flash‐frozen using liquid nitrogen. Tumor samples from NS and FSP‐RZ‐BPH groups were subjected to RNA‐seq analysis (GENEWIZ, Suzhou, China). The quantification of gene expression and subsequent differential expression analysis were conducted utilizing the DESeq2 Bioconductor package. Functionally associated Gene Ontology (GO) terms for biological processes were scrutinized using GOSeq (v1.34.1), while Kyoto Encyclopedia of Genes and Genomes (KEGG) enrichment analysis drew upon the pertinent database (http://en.wikipedia.org/wiki/KEGG). Additionally, GO network analysis of significantly upregulated genes within tumors was undertaken through the application of Cytoscape software.

### Tumor Cell Killing Assay

The splenocytes obtained from the aforementioned MFC prevention model were used for tumor killing assay. The analysis consisted of two levels. First, splenocytes from different groups were used to kill MFC tumor cells. Second, splenocytes from NS and FSP‐RZ‐BPH groups were used to kill four different tumor cells: MFC gastric cancer cells, 4T1 breast cancer cells, B16F10 melanoma cells and MC38 colorectal cancer cells. Specifically, add 1 µM CFSE into 1 × 10^6^ ml^−1^ tumor cells for incubating at 37 °C in the dark for 8–10 min. Then add ten‐folds the volume of PBS to stop the reaction. After centrifuging at 500 g for 3 min, discard the supernatant to obtain the tumor cells as target cells. Splenocytes were used as effector cells to be incubated with target cells by the effect‐to‐target ratio of 10:1 at 37 °C for 4–6 h. Afterward, the co‐incubated cells were centrifuged at 500 g for 5 min, and the supernatant was discarded. Add 100 ng ml^−1^ PI solution into the collected cells for incubating at room temperature in the dark for 10 min, and then analyze using a BeckMan CytoFlex.

### Safety Analysis

All the 615 mice were sacrificed on D10 after the last administration. Then the main organs including the heart, liver, spleen, lung, and kidney were dissected and fixed in 4% formaldehyde. Paraffin‐embedded slides (4 µm in thickness) were prepared and stained with hematoxylin and eosin (H&E) for histological analysis under optical microscopy (DM5000, Leica, Germany). Besides, the serum was collected on D10 after the last administration to assay the liver and kidney function by testing the levels of AST, ALT, ALP and BUN, CREA.

### Statistical Analysis

Statistical analysis was performed using GraphPad Prism 8.0.2 statistical software (San Diego, CA, USA). All results were presented as mean ± SEM for at least three independent experiments and *P*‐values < 0.05 were considered to indicate statistical significance. For tumor burden comparisons, p‐values were calculated by two‐tailed unpaired Student's t‐tests or two‐way ANOVA and Tukey post‐test and correction as indicated. For survival studies, log‐rank (Mantel–Cox) tests were used. Figures were designed in Adobe Illustrator. For FACS studies and other experiments, student's t‐tests were used. Figures were designed in Adobe Illustrator.

## Conflict of Interest

The authors declare no conflict of interest.

## Author Contributions

Y.K., B.L., and Q.L. conceived and designed the experiments. Y.K. performed the experiments. K.X., L.L., A.C., J.S., J.Z., Y.T., D.Z., L.C., and Y.C. assisted in the experiments and data analysis. Y.K. and Q.L. prepared the manuscript. B.L. and Q.L. supervised the project.

## Supporting information

Supporting Information

## Data Availability

The data that support the findings of this study are available from the corresponding author upon reasonable request.

## References

[1] Z. Hu , P. A. Ott , C. J. Wu , Nat. Rev. Immunol. 2018, 18, 168. 29226910 10.1038/nri.2017.131 PMC6508552

[2] a) T. N. Schumacher , R. D. Schreiber , Science 2015, 348, 69; 25838375 10.1126/science.aaa4971

[3] G. Garrido , B. Schrand , A. Levay , A. Rabasa , A. Ferrantella , D. M. Da Silva , F. D'Eramo , K. A. Marijt , Z. Zhang , D. Kwon , M. Kortylewski , W. M. Kast , V. Dudeja , T. van Hall , E. Gilboa , Cancer Immunol. Res. 2020, 8, 856. 32295785 10.1158/2326-6066.CIR-20-0020 PMC7339786

[4] a) A. Poole , V. Karuppiah , A. Hartt , J. N. Haidar , S. Moureau , T. Dobrzycki , C. Hayes , C. Rowley , J. Dias , S. Harper , K. Barnbrook , M. Hock , C. Coles , W. Yang , M. Aleksic , A. B. Lin , R. Robinson , J. D. Dukes , N. Liddy , M. Van der Kamp , G. D. Plowman , A. Vuidepot , D. K. Cole , A. D. Whale , C. Chillakuri , Nat. Commun. 2022, 13, 5333; 36088370 10.1038/s41467-022-32811-1 PMC9464187

[5] a) C. C. Smith , S. R. Selitsky , S. Chai , P. M. Armistead , B. G. Vincent , J. S. Serody , Nat. Rev. Cancer 2019, 19, 465; 31278396 10.1038/s41568-019-0162-4 PMC6874891

[6] a) P. A. Ott , S. Hu‐Lieskovan , B. Chmielowski , R. Govindan , A. Naing , N. Bhardwaj , K. Margolin , M. M. Awad , M. D. Hellmann , J. J. Lin , T. Friedlander , M. E. Bushway , K. N. Balogh , T. E. Sciuto , V. Kohler , S. J. Turnbull , R. Besada , R. R. Curran , B. Trapp , J. Scherer , A. Poran , D. Harjanto , D. Barthelme , Y. S. Ting , J. Z. Dong , Y. Ware , Y. Huang , Z. Huang , A. Wanamaker , L. D. Cleary , et al., Cell 2020, 183, 347; 33064988 10.1016/j.cell.2020.08.053

[7] M. M. Awad , R. Govindan , K. N. Balogh , D. R. Spigel , E. B. Garon , M. E. Bushway , A. Poran , J. H. Sheen , V. Kohler , E. Esaulova , J. Srouji , S. Ramesh , R. Vyasamneni , B. Karki , T. E. Sciuto , H. Sethi , J. Z. Dong , M. A. Moles , K. Manson , M. S. Rooney , Z. S. Khondker , M. DeMario , R. B. Gaynor , L. Srinivasan , Cancer Cell 2022, 40, 1010. 36027916 10.1016/j.ccell.2022.08.003

[8] a) S. Turajlic , K. Litchfield , H. Xu , R. Rosenthal , N. McGranahan , J. L. Reading , Y. N. S. Wong , A. Rowan , N. Kanu , M. Al Bakir , T. Chambers , R. Salgado , P. Savas , S. Loi , N. J. Birkbak , L. Sansregret , M. Gore , J. Larkin , S. A. Quezada , C. Swanton , Lancet Oncol. 2017, 18, 1009; 28694034 10.1016/S1470-2045(17)30516-8

[9] K. Ando , Y. Nakamura , H. Kitao , M. Shimokawa , D. Kotani , H. Bando , T. Nishina , T. Yamada , S. Yuki , Y. Narita , H. Hara , T. Ohta , T. Esaki , Y. Hamamoto , K. Kato , Y. Yamamoto , K. Minashi , K. Ohtsubo , N. Izawa , H. Kawakami , T. Kato , T. Satoh , N. Okano , A. Tsuji , K. Yamazaki , T. Yoshino , Y. Maehara , E. Oki , Br. J. Cancer 2023, 129, 1032. 37532830 10.1038/s41416-023-02378-9 PMC10491760

[10] D. T. Le , J. N. Durham , K. N. Smith , H. Wang , B. R. Bartlett , L. K. Aulakh , S. Lu , H. Kemberling , C. Wilt , B. S. Luber , F. Wong , N. S. Azad , A. A. Rucki , D. Laheru , R. Donehower , A. Zaheer , G. A. Fisher , T. S. Crocenzi , J. J. Lee , T. F. Greten , A. G. Duffy , K. K. Ciombor , A. D. Eyring , B. H. Lam , A. Joe , S. P. Kang , M. Holdhoff , L. Danilova , L. Cope , C. Meyer , et al., Science 2017, 357, 409. 28596308 10.1126/science.aan6733 PMC5576142

[11] F. J. Lowery , S. Krishna , R. Yossef , N. B. Parikh , P. D. Chatani , N. Zacharakis , M. R. Parkhurst , N. Levin , S. Sindiri , A. Sachs , K. J. Hitscherich , Z. Yu , N. R. Vale , Y. C. Lu , Z. Zheng , L. Jia , J. J. Gartner , V. K. Hill , A. R. Copeland , S. K. Nah , R. V. Masi , B. Gasmi , S. Kivitz , B. C. Paria , M. Florentin , S. P. Kim , K. I. Hanada , Y. F. Li , L. T. Ngo , S. Ray , et al., Science 2022, 375, 877. 35113651 10.1126/science.abl5447 PMC8996692

[12] Q. Liu , Y. Chu , J. Shao , H. Qian , J. Yang , H. Sha , L. Cen , M. Tian , Q. Xu , F. Chen , Y. Yang , W. Wang , K. Wang , L. Yu , J. Wei , B. Liu , Adv. Sci. 2022, 10, e2203298. 10.1002/advs.202203298 PMC9811442 36351249

[13] T. F. Gajewski , H. Schreiber , Y. X. Fu , Nat. Immunol. 2013, 14, 1014. 24048123 10.1038/ni.2703 PMC4118725

[14] a) X. Huang , J. Pan , F. Xu , B. Shao , Y. Wang , X. Guo , S. Zhou , Adv. Sci. 2021, 8, 2003572; 10.1002/advs.202003572 PMC8025040 33854892

[15] a) A. Ribas , T. Medina , J. M. Kirkwood , Y. Zakharia , R. Gonzalez , D. Davar , B. Chmielowski , K. M. Campbell , R. Bao , H. Kelley , A. Morris , D. Mauro , J. E. Wooldridge , J. J. Luke , G. J. Weiner , A. M. Krieg , M. M. Milhem , Cancer Discov 2021, 11, 2998; 34326162 10.1158/2159-8290.CD-21-0425 PMC8799774

[16] a) M. Lv , M. Chen , R. Zhang , W. Zhang , C. Wang , Y. Zhang , X. Wei , Y. Guan , J. Liu , K. Feng , M. Jing , X. Wang , Y. C. Liu , Q. Mei , W. Han , Z. Jiang , Cell Res. 2020, 30, 966; 32839553 10.1038/s41422-020-00395-4 PMC7785004

[17] a) L. Zhang , J. Zhao , X. Hu , C. Wang , Y. Jia , C. Zhu , S. Xie , J. Lee , F. Li , D. Ling , Adv. Mater. 2022, 34, 2206915; 10.1002/adma.202206915 35986645

[18] L. Zhang , X. Wei , Z. Wang , P. Liu , Y. Hou , Y. Xu , H. Su , M. D. Koci , H. Yin , C. Zhang , Cell Rep. 2023, 42, 112185. 36857187 10.1016/j.celrep.2023.112185

[19] E. C. Cheung , K. H. Vousden , Nat. Rev. Cancer 2022, 22, 280. 35102280 10.1038/s41568-021-00435-0

[20] a) D. Seliktar , Science 2012, 336, 1124; 22654050 10.1126/science.1214804

[21] Q. Liu , Y. Chu , J. Shao , H. Qian , J. Yang , H. Sha , L. Cen , M. Tian , Q. Xu , F. Chen , Y. Yang , W. Wang , K. Wang , L. Yu , J. Wei , B. Liu , Adv. Sci. 2022, 10, e2203298. 10.1002/advs.202203298 PMC9811442 36351249

[22] R. Kuai , L. J. Ochyl , K. S. Bahjat , A. Schwendeman , J. J. Moon , Nat. Mater. 2017, 16, 489. 28024156 10.1038/nmat4822 PMC5374005

[23] E. Forte , B. Perkins , A. Sintou , H. S. Kalkat , A. Papanikolaou , C. Jenkins , M. Alsubaie , R. A. Chowdhury , T. M. Duffy , D. A. Skelly , J. Branca , M. Bellahcene , M. D. Schneider , S. E. Harding , M. B. Furtado , F. S. Ng , M. G. Hasham , N. Rosenthal , S. Sattler , Circulation 2021, 143, 821. 33297741 10.1161/CIRCULATIONAHA.120.044581 PMC7899721

[24] a) K. Wang , G. Buschle‐Diller , Y. Wu , J. Appl. Polym. Sci. 2014, 131, 20;

[25] D. M. Francis , M. P. Manspeaker , A. Schudel , L. F. Sestito , M. J. O'Melia , H. T. Kissick , B. P. Pollack , E. K. Waller , S. N. Thomas , Sci. Transl. Med. 2020, 12, eaay3575. 32998971 10.1126/scitranslmed.aay3575 PMC8377700

[26] Y. Chu , L. Qian , Y. Ke , X. Feng , X. Chen , F. Liu , L. Yu , L. Zhang , Y. Tao , R. Xu , J. Wei , B. Liu , Q. Liu , J Nanobiotechnology 2022, 20, 190. 35418151 10.1186/s12951-022-01397-7 PMC9006542

[27] Q. Zou , X. Wang , D. Ren , B. Hu , G. Tang , Y. Zhang , M. Huang , R. K. Pai , D. D. Buchanan , A. K. Win , P. A. Newcomb , W. M. Grady , H. Yu , Y. Luo , J Immunother Cancer 2021, 9, e002671. 34548385 10.1136/jitc-2021-002671 PMC8458312

[28] G. Cafri , J. J. Gartner , T. Zaks , K. Hopson , N. Levin , B. C. Paria , M. R. Parkhurst , R. Yossef , F. J. Lowery , M. S. Jafferji , T. D. Prickett , S. L. Goff , C. T. McGowan , S. Seitter , M. L. Shindorf , A. Parikh , P. D. Chatani , P. F. Robbins , S. A. Rosenberg , J. Clin. Invest. 2020, 130, 5976. 33016924 10.1172/JCI134915 PMC7598064

[29] Q. Ni , F. Zhang , Y. Liu , Z. Wang , G. Yu , B. Liang , G. Niu , T. Su , G. Zhu , G. Lu , L. Zhang , X. Chen , Sci. Adv. 2020, 6, eaaw6071. 32206706 10.1126/sciadv.aaw6071 PMC7080439

[30] M. O. Meneveau , G. R. Petroni , E. P. Salerno , K. T. Lynch , M. Smolkin , E. Woodson , K. A. Chianese‐Bullock , W. C. Olson , D. Deacon , J. W. Patterson , W. W. Grosh , C. L. Slingluff , J Immunother Cancer 2021, 9, e002214. 34035112 10.1136/jitc-2020-002214 PMC8154977

[31] C. Jin , J. Ma , M. Ramachandran , D. Yu , M. Essand , Nat. Biomed. Eng. 2022, 6, 830. 35379957 10.1038/s41551-022-00875-5 PMC9288934

[32] A. Atalis , J. B. Dixon , K. Roy , Adv. Healthcare Mater. 2021, 10, e2001899. 10.1002/adhm.202001899 PMC9211062 33928762

