# Supplementary material for: A Thermosensitive Bi‐Adjuvant Hydrogel Triggers Epitope Spreading to Promote the Anti‐Tumor Efficacy of Frameshift Neoantigens
Source: Adv Sci (Weinh). 2024 Feb 2;11(14):2306889. doi: 10.1002/advs.202306889 (PMC11005695; doi:10.1002/advs.202306889)
Supplement: Supplementary file 1 — Supporting Information [file ADVS-11-2306889-s001.pdf]

ADVANCED SCIENCE
Open Access

Supporting Information

for *Adv. Sci.*, DOI 10.1002/advs.202306889

A Thermosensitive Bi-Adjuvant Hydrogel Triggers Epitope Spreading to Promote the Anti-Tumor Efficacy of Frameshift Neoantigens

Yaohua Ke, Kai Xin, Yaping Tao, Lin Li, Aoxing Chen, Jingyi Shao, Junmeng Zhu, Dinghu Zhang, Lanqi Cen, Yanhong Chu, Lixia Yu, Baorui Liu and Qin Liu**

Supporting Information

A thermosensitive bi-adjuvant hydrogel triggers epitope spreading to promote the anti-tumor efficacy of frameshift neoantigens

Yaohua Ke[1], Kai Xin[2], Yaping Tao[1], Lin Li[2], Aoxing Chen[2], Jingyi Shao[2], Junmeng Zhu[1], Dinghu Zhang[3], Lanqi Cen[1], Yanhong Chu[1], Lixia Yu[1], Baorui Liu[1,2], Qin Liu[1,2]**

a

No.MFC	Gene	Mutation	MFC bound to H-2K^k	peptide affinity(nm)	%Rank
MFC 1	Olfr1133	p.N196D	NEIVMFTLI	7.7915	0.0085
MFC 2	Olfr472	p.L90M	MELLGHGMV	17.8102	0.0369
MFC 3	Hsd3b1	p.A232T	VENVAWTHI	19.6188	0.0456
MFC 4	Cers3	p.S223N	LEMNFYWSL	39.1041	0.1392
MFC 5	Dars2	p.D382N	IEFIRKFAV	39.9872	0.143
MFC 6	Cers3	p.S223N	LEMNFYWSLV	14.2039	0.023

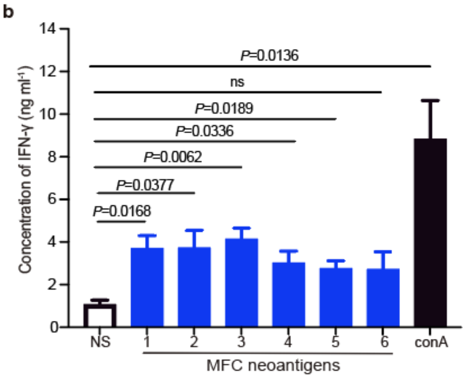

Figure S1. a, Amino acid sequence of MFCs and their affinity to H-2K^K. **b,** Concentration of IFN-γ secreted by splenocytes of 615 mice stimulated by MFCs (n=3). The error bars represented mean ± SEM. *P*-values were calculated by two-tailed unpaired Student's t-tests. ns represented *P*> 0.05.

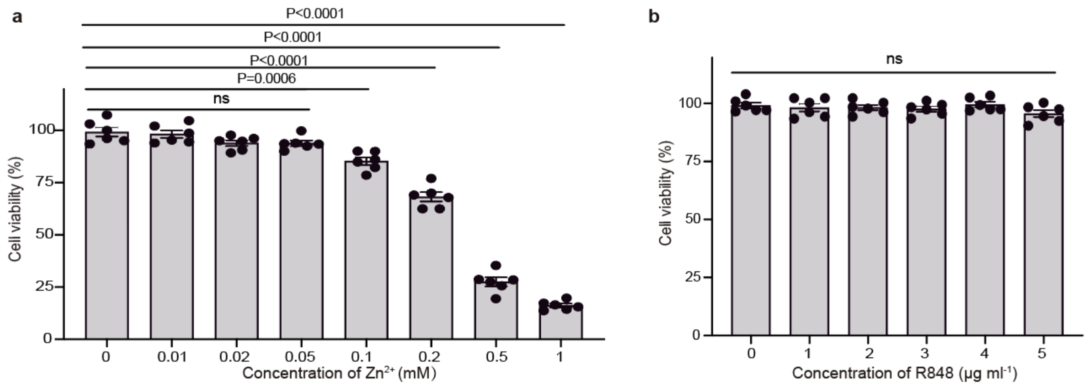

Figure. S2. a, Cell viability of HUVECs after treated with different concentrations of Zn^{2+} for 24 h (n=6). **b,** Cell viability of HUVECs after treated with different concentrations of R848 for 24 h (n=6). The error bars represented mean ± SEM. *P*-values were calculated by two-tailed unpaired Student's t-tests. ns represented *P* > 0.05.

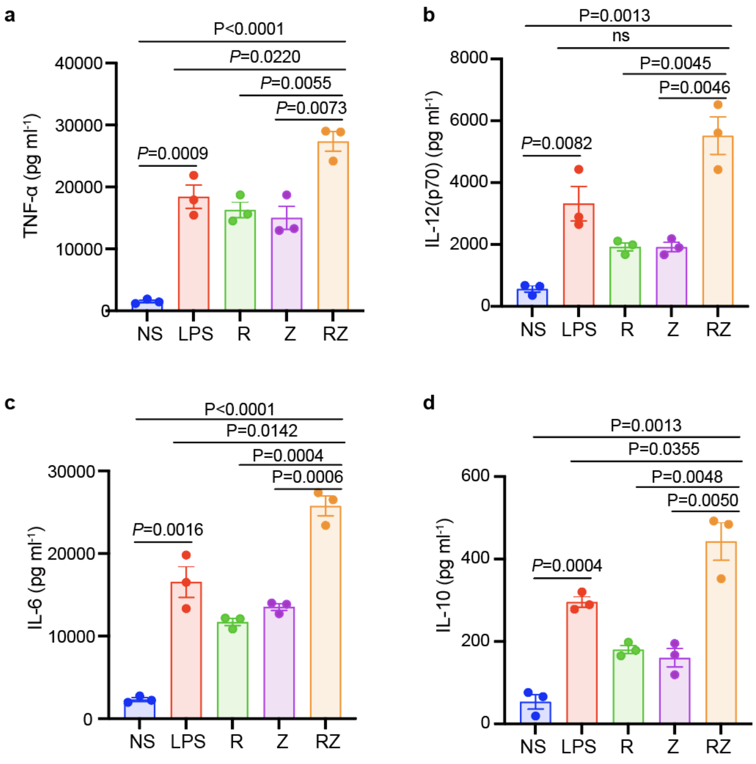

Figure S3. a-d, the levels of TNF-α, IL-12(p70), IL-6, and IL-10 secreted from BMDCs after different treatment (n=3). The error bars represented mean ± SEM. *P*-values were calculated by two-tailed unpaired Student's t-tests. ns represented *p* > 0.05.

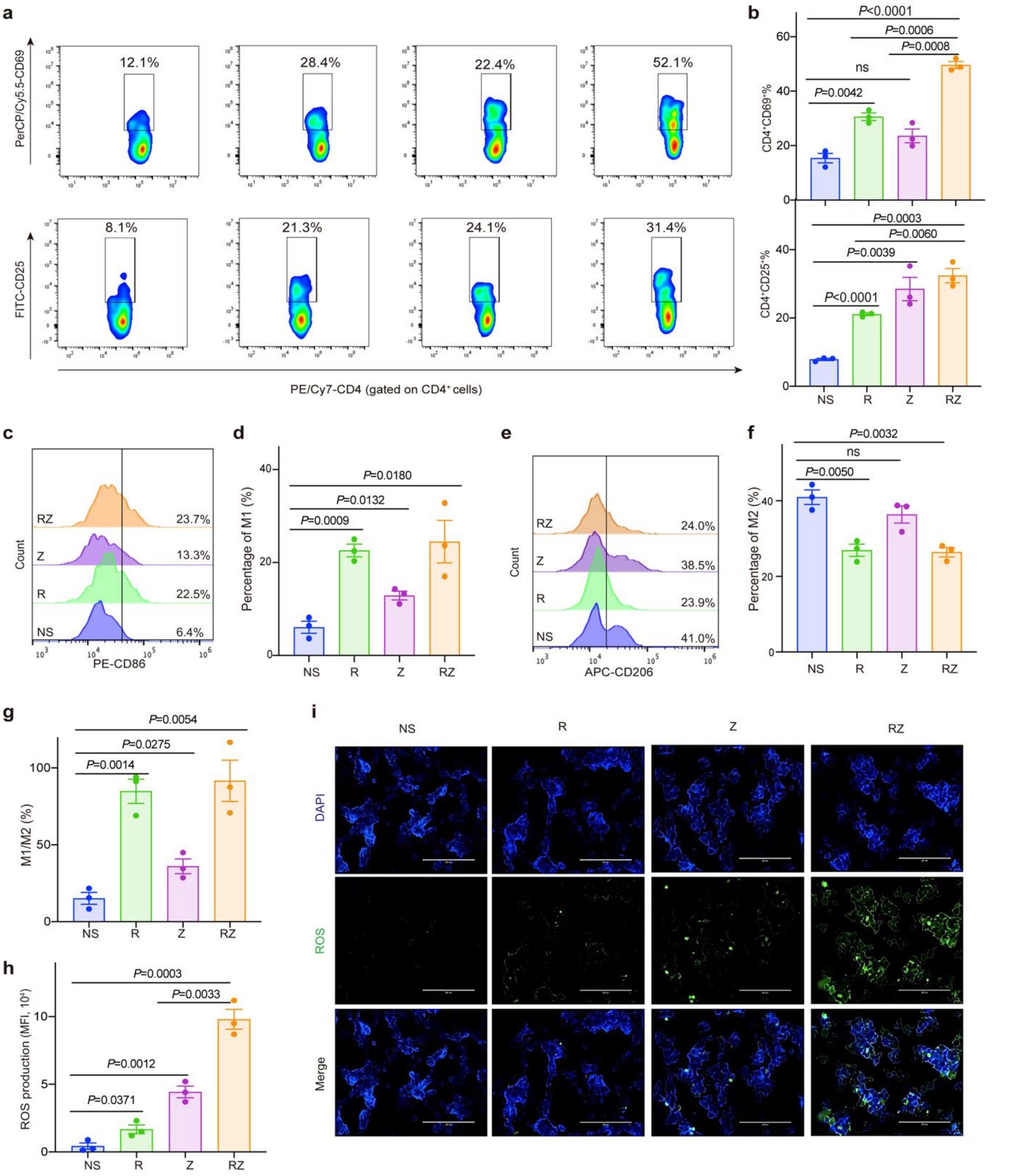

Figure S4. The pro-inflammatory effect of R848 + Zn^{2+} (RZ) in $CD4^+$ T cells, macrophages and ROS production. a, Representative flow cytometry images of $CD4^+CD25^+$ T cells and $CD4^+CD69^+$ T cells after co-incubation with RZ in vitro for 48 h. **b,** The percentage $CD4^+CD25^+$ T cells and $CD4^+CD69^+$ T cells (n=3). **c,** Representative flow cytometry images of PE-CD86 expression on RAW264.7 cells after co-incubation with RZ in vitro for 48 h. **d,** Percentage of M1

macrophages (F4/80$^+$CD11b$^+$CD86$^+$ RAW264.7 cells) (n=3). **e,** Representative flow cytometry images of APC-CD206 expression on RAW264.7 cells after co-incubation with RZ in vitro for 48 h. **f,** Percentage of M2 macrophages (F4/80$^+$CD11b$^+$CD206$^+$ RAW264.7 cells) (n=3). **g,** Percentage of M1/M2 (n=3). **h**, ROS production on MFC cells after co-incubation with RZ in vitro for 48 h. Average DCF fluorescence intensity represented the levels of ROS. **i,** Fluorescence images of ROS production (green) in MFC cells after different treatments (The scale bar is 200 μm). The error bars represented mean ± SEM. *P*-values were calculated by two-tailed unpaired Student's t-tests. ns represented $p > 0.05$.

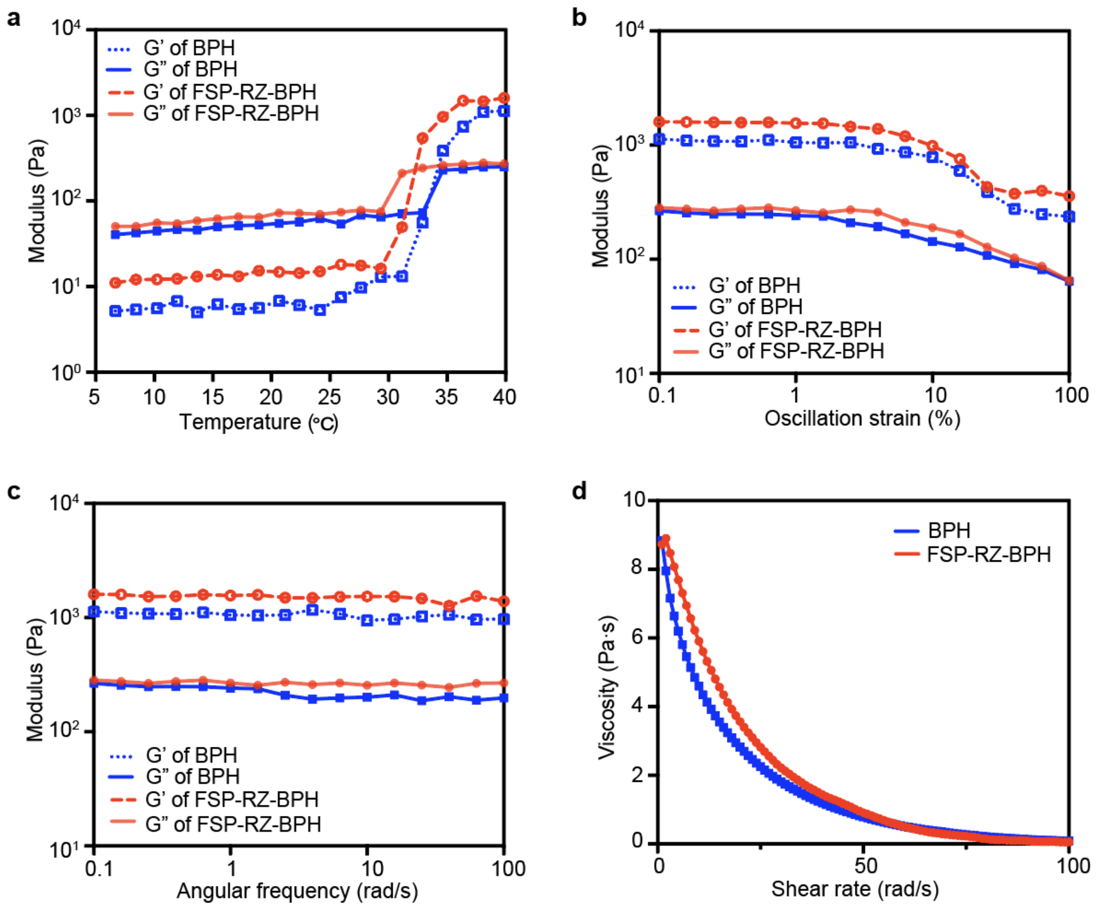

Figure S5. Rheological measurements of BPH and FSP-RZ-BPH. Evolution of storage modulus (G') and loss modulus (G'') under different **a,** Temperature (γ = 1 %, ε = 10 rad/s, heating rate 0.5℃/min); **b,** Oscillation strains (ε = 10 rad/s, T= 37℃) and **c,** Angular frequency (γ

= 1 %, T= 37℃). **d,** Viscosity of hydrogel versus shear rate (rad/s, T= 37℃). All the samples were measured with a rotating rheometer.

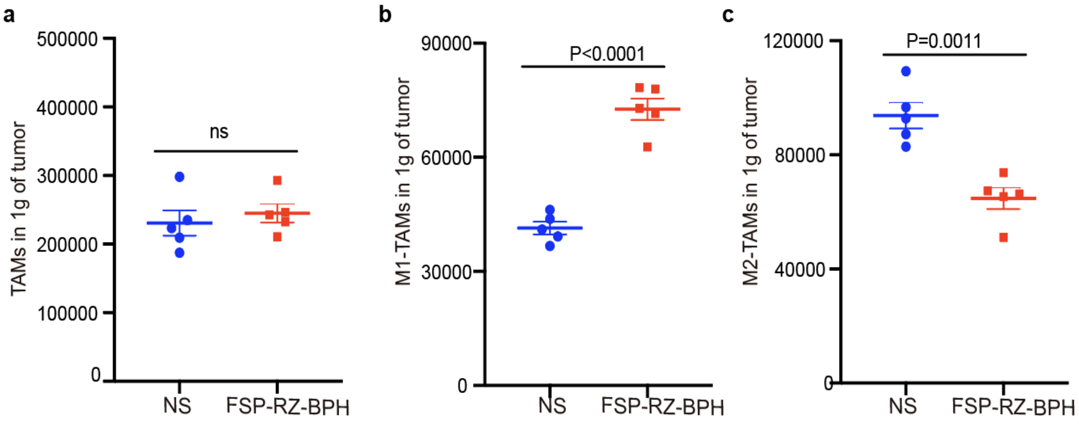

Figure S6. a, Total numbers of tumor-associated macrophages (TAMs) in 1g of tumors in NS and FSP-RZ-BPH groups (n=5). **b,** Numbers of M1-TAMs in 1g of tumors in NS and FSP-RZ-BPH groups (n=5). **c,** Numbers of M2-TAMs in 1g of tumors in NS and FSP-RZ-BPH groups (n=5). The error bars represented mean ± SEM. P-values were calculated by two-tailed unpaired Student’s t-tests. ns represented $p > 0.05$.

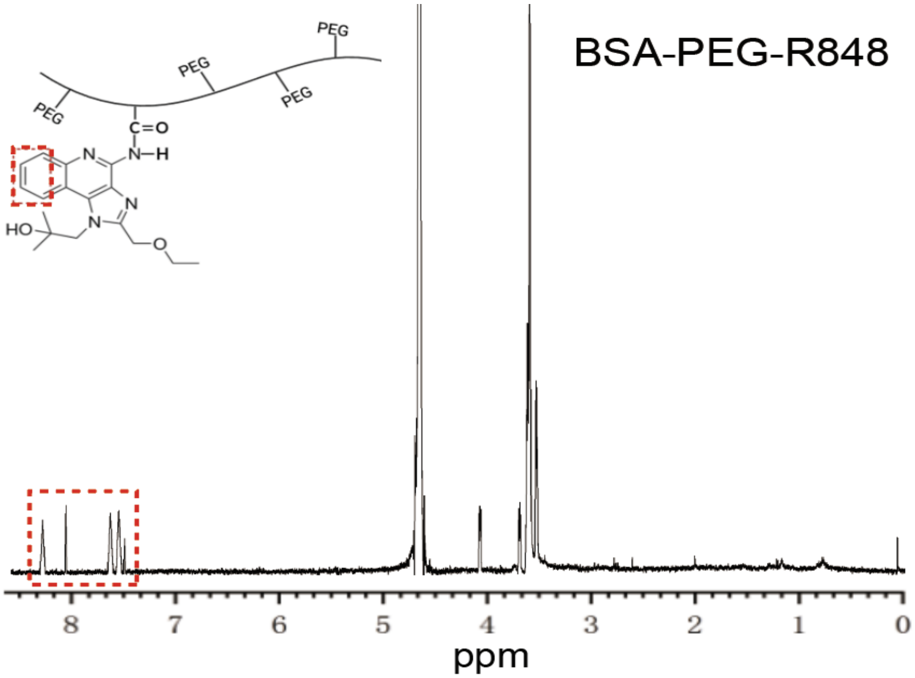

Figure S7. H^1-NMR spectra of BSA–PEG-R848.

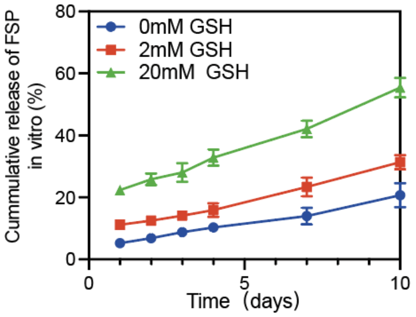

Figure S8. Cumulative release of FSP from FSP-RZ-BPH in different concentrations of GSH in vitro (n = 3).

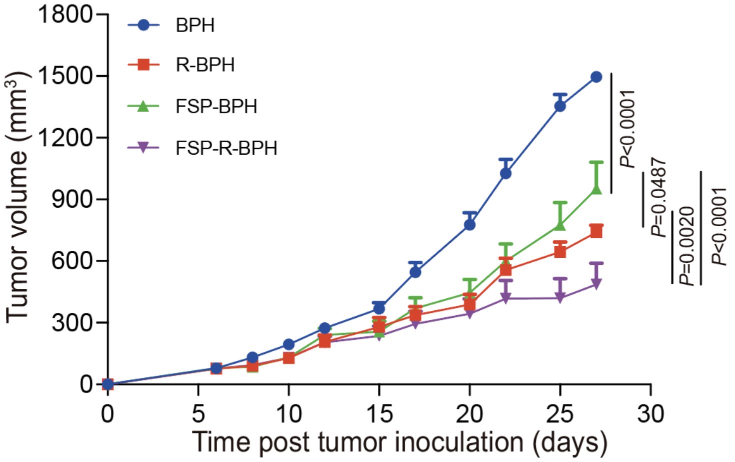

Figure S9. Average tumor-growth curves of 615 mice bearing MFC gastric cancer in BPH, R-BPH, FSP-BPH and FSP-R-BPH groups in 27 days (n = 6). The error bars represented mean ± SEM. *P*-values were calculated by two-way ANOVA and Tukey post-test and correction.

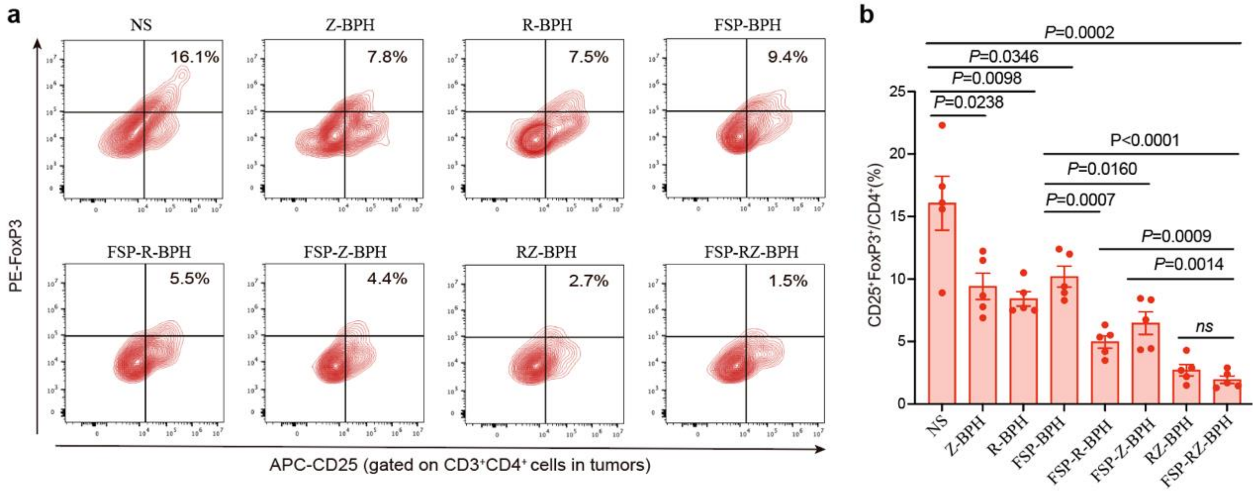

Figure S10. a, Representative flow cytometry images of Tregs ($CD3^{+}CD4^{+}CD25^{+}FoxP3^{+}$) in tumors in different groups. **b,** Percentage of Tregs in tumors in different groups (n=5). The error bars represented mean ± SEM. P-values were calculated by two-tailed unpaired Student's t-tests. ns represented p > 0.05.

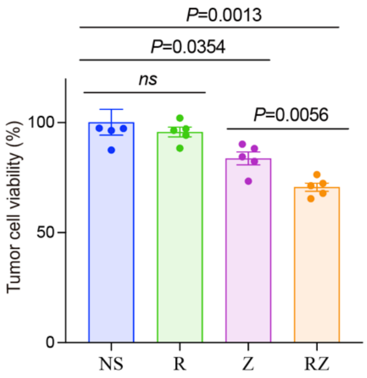

Figure S11. Cell viability of MFC cells after different treatment for 48 h (n=5). The error bars represented mean ± SEM. P-values were calculated by two-tailed unpaired Student's t-tests. ns represented P > 0.05.

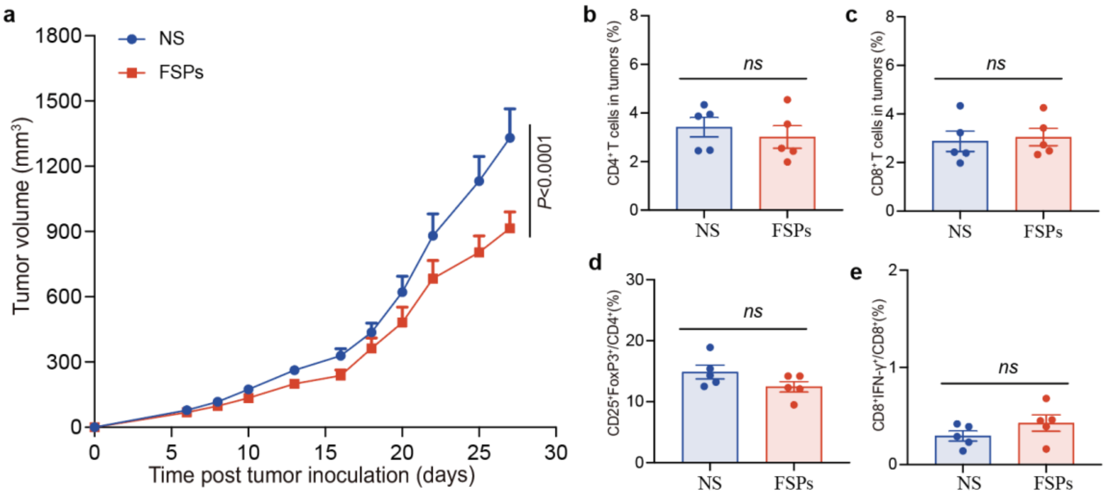

Figure S12. a, Average tumor-growth curves of 615 mice bearing MFC gastric cancer in NS and FSPs groups (n = 6). **b,** Percentage of $CD4^+$ T cells in tumors in NS and FSPs groups (n=5). **c,** Percentage of $CD8^+$ T cells in tumors in NS and FSPs groups (n=5). **d,** Percentage of Tregs ($CD3^+CD4^+CD25^+FoxP3^+$) in tumors in NS and FSPs groups (n=5). **e,** Percentage of IFN-γ^+CD8^+ T cells in tumors in NS and FSPs groups (n=5). The error bars represented mean ± SEM. *P*-values were calculated by two-way ANOVA and Tukey post-test and correction (a) or two-tailed unpaired Student's t-tests (b, c, d, e). ns represented $p > 0.05$.

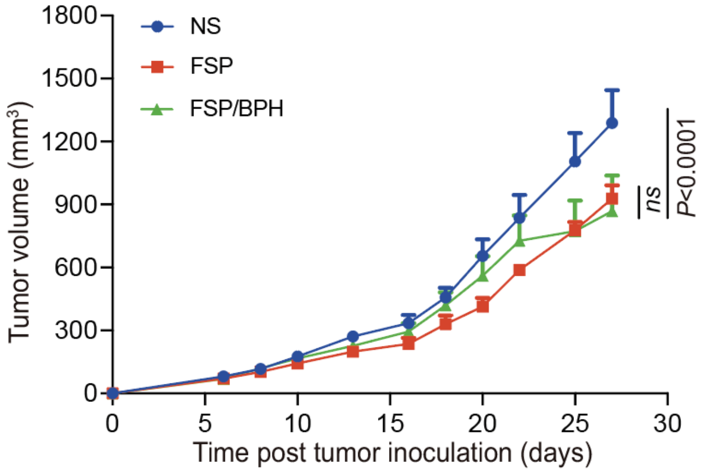

Figure S13. Average tumor-growth curves of 615 mice bearing MFC gastric cancer in BPH, R-BPH, FSP-BPH and FSP-R-BPH groups in 27 days (n = 6). The error bars represented mean ± SEM. *P*-values were calculated by two-way ANOVA and Tukey post-test and correction. ns represented $p > 0.05$.

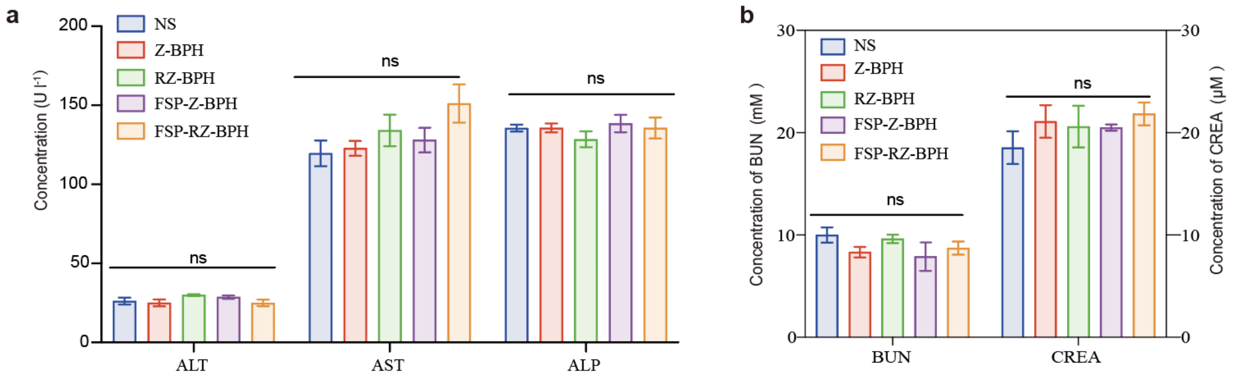

Figure S14. a, Liver function (AST, ALT and ALP) and **b,** Kidney function (BUN and CREA) analysis of 615 mice in different groups (n = 7). The sera were collected on D23 (n=5). *P*-values were calculated by two-tailed unpaired Student's t-tests. ns represented p > 0.05.

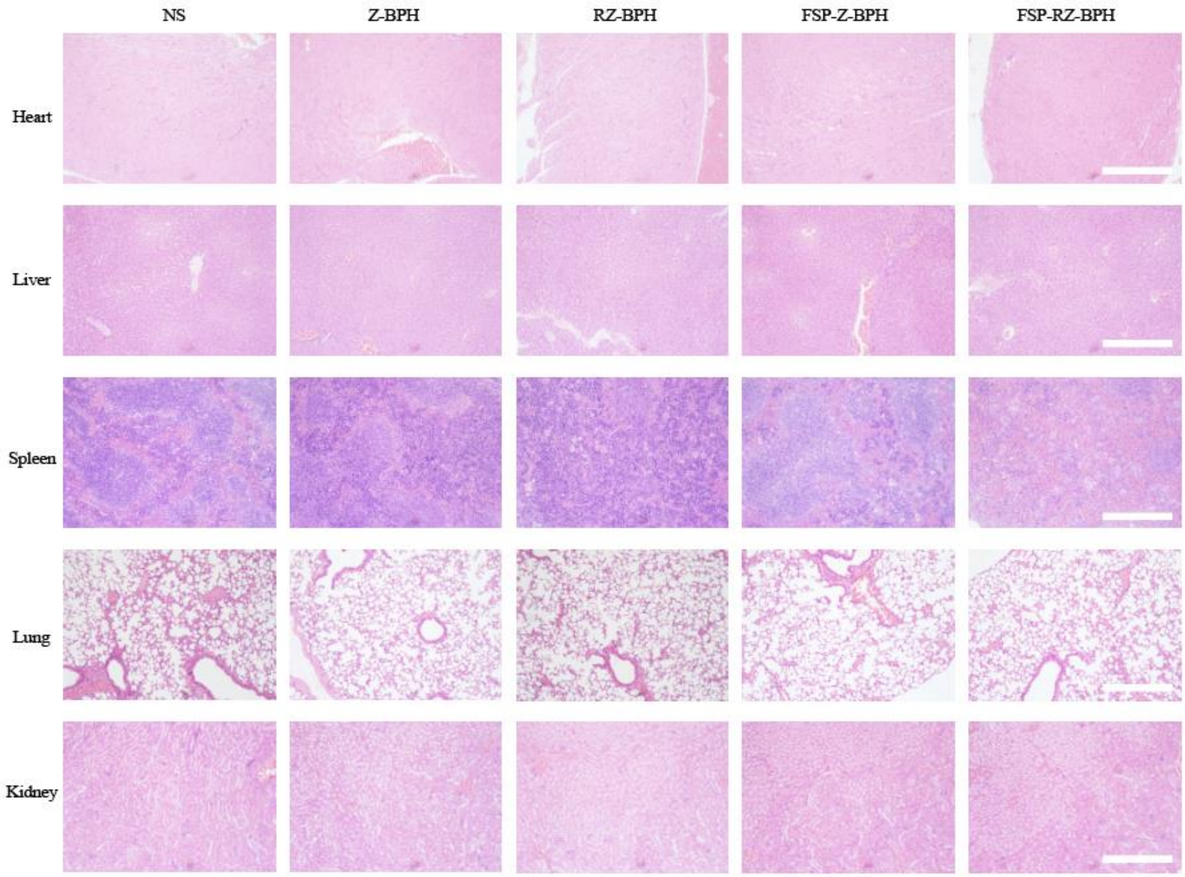

Figure S15. H&E staining of heart, liver, spleen, lung, and kidney in 615 mouse MFC tumour suppression experiment model on D23 (10 days after last treatments).
